# The Rac GTP Exchange Factor TIAM-1 Acts with CDC-42 and the Guidance Receptor UNC-40/DCC in Neuronal Protrusion and Axon Guidance

**DOI:** 10.1371/journal.pgen.1002665

**Published:** 2012-04-26

**Authors:** Rafael S. Demarco, Eric C. Struckhoff, Erik A. Lundquist

**Affiliations:** Programs in Genetics and Molecular, Cellular, and Developmental Biology, Department of Molecular Biosciences, University of Kansas, Lawrence, Kansas, United States of America; University of California San Diego, United States of America

## Abstract

The mechanisms linking guidance receptors to cytoskeletal dynamics in the growth cone during axon extension remain mysterious. The Rho-family GTPases Rac and CDC-42 are key regulators of growth cone lamellipodia and filopodia formation, yet little is understood about how these molecules interact in growth cone outgrowth or how the activities of these molecules are regulated in distinct contexts. UNC-73/Trio is a well-characterized Rac GTP exchange factor in *Caenorhabditis elegans* axon pathfinding, yet UNC-73 does not control CED-10/Rac downstream of UNC-6/Netrin in attractive axon guidance. Here we show that *C. elegans* TIAM-1 is a Rac-specific GEF that links CDC-42 and Rac signaling in lamellipodia and filopodia formation downstream of UNC-40/DCC. We also show that TIAM-1 acts with UNC-40/DCC in axon guidance. Our results indicate that a CDC-42/TIAM-1/Rac GTPase signaling pathway drives lamellipodia and filopodia formation downstream of the UNC-40/DCC guidance receptor, a novel set of interactions between these molecules. Furthermore, we show that TIAM-1 acts with UNC-40/DCC in axon guidance, suggesting that TIAM-1 might regulate growth cone protrusion via Rac GTPases in response to UNC-40/DCC. Our results also suggest that Rac GTPase activity is controlled by different GEFs in distinct axon guidance contexts, explaining how Rac GTPases can specifically control multiple cellular functions.

## Author Summary

Axons extend great distances to make precise synaptic connections in the developing nervous system. Axons are guided to their targets by the growth cone, a dynamic structure at the axon distal tip that senses extracellular cues telling the axon where to go. In response to guidance cues, growth cones alter their shape and motility resulting in outgrowth and turning. The cytoskeleton (actin and microtubules) underlies growth cone motility and guidance. The signaling mechanisms linking guidance receptors to cytoskeletal change remain mysterious. Here, we define a new signaling mechanism downstream of the guidance receptor UNC-40/DCC involving the GTPases CDC-42 and Rac, which have long been known to control growth cone protrusion. We show that CDC-42 and Rac act in a linear pathway in axon guidance; CDC-42 acts upstream of the GTPase regulatory molecule TIAM-1, which is a GTP exchange factor specific for Rac and which activates Rac signaling. We also show that TIAM-1 acts with UNC-40/DCC signaling in protrusion and axon guidance. Our results imply that Rac GTPase function in axon guidance is complex and that distinct GEFs (TIAM-1 and UNC-73/Trio) might control Rac GTPases in different aspects of axon guidance.

## Introduction

In a developing nervous system, axonal growth happens via extension and migration of the growth cone, an actin-rich neuronal structure from which filopodial and lamellipodial structures emanate in order to sense the surrounding environment for appropriate guidance cues [1]–[5]. Guidance receptors such as Deleted in Colorectal Carcinoma (DCC) and Roundabout (ROBO) are present in the leading edge of the growth cone and are activated upon ligand binding (such as Netrin and Slit, respectively) [6]–[10]. Attractive and repellent guidance systems guide the growth cone toward its final target, presumably by affecting the protrusiveness of filopodial and lamellipodial structures in the growth cone [8], [11]. The proper control of the actin cytoskeleton is crucial for the development, maintenance and dynamics of these filopodia and lamellipodia. Failure in the proper extension of axons during development is associated with several neurological disorders, including autism spectrum disorders and dyslexia [12], [13].

The intracellular signaling mechanisms linking guidance receptors to cytoskeletal alterations are an intense area of study, and many remain to be elucidated. Rac GTPases, members of the Rho subfamily of small GTPases (which also includes Rho and Cdc-42), have been well established as modulators of the actin cytoskeleton [14] and axon guidance [15]. Rac GTPases and their downstream effectors modulate filopodia and lamellipodia morphology and dynamics during axon pathfinding in vitro in mammalian cell culture, as well as in vivo in the fruit fly *Drosophila melanogaster* and the nematode *Caenorhabditis elegans* [15]–[18]. Rac GTPases act on the actin cytoskeleton via diverse downstream effectors such as the actin-filament nucleator Arp2/3 complex, the severing protein Cofilin, the Pak and LIM kinases, and the actin binding protein UNC-115/abLIM [19]–[23]. In *C. elegans* growth cone development and axon pathfinding, the two redundant Rac GTPases CED-10/Rac1 and MIG-2/RhoG act via two distinct pathways to activate the Arp2/3 complex: CED-10/Rac1 acts with WVE-1/Wave, while MIG-2/RhoG acts with WSP-1/Wasp [24]. CED-10/Rac1 is also known to act with the actin-binding protein UNC-115/abLIM via the Receptor for Activated C Kinase (RACK-1) [25].

Although much is known about the downstream effectors of the Rac GTPases in axon development, less is known about pathways upstream of Rac GTPases that control their activity in vivo. Rac GTPases switch between an inactive, GDP-bound state, and an active, GTP-bound state. Rac GTPase activation can be reached with the aid of a Guanine-nucleotide Exchange Factor (GEF), which catalyzes the exchange of GTP for GDP [26]. The *C. elegans* genome encodes 19 predicted Dbl-homology (DH) GEF proteins (Wormbase). UNC-73/Trio is a well-characterized GEF for MIG-2/RhoG and CED-10/Rac1 in *C. elegans* axon pathfinding [27]–[30]. A mutation that specifically disrupts specifically the RacGEF activity of UNC-73/Trio (*rh40*) disrupts axon pathfinding [30]. However, pathfinding defects of *mig-2; ced-10* rac double mutants are more severe than *unc-73(rh40)* [28], indicating that additional GEFs act with Rac GTPases to control axon pathfinding.

Here we show that TIAM-1 is a Rac GEF similar to mammalian T-cell lymphoma Invasion and Metastasis Factor 1 (Tiam1) [31] and Drosophila Still Life (Sif) [32]. Previous results suggest that the UNC-73/Trio Rac GEF does not act with CED-10/Rac1 downstream of UNC-40/DCC in neuronal protrusion [9]. Here we show that the TIAM-1 Rac GEF acts downstream of the UNC-40/DCC netrin receptor in neuronal protrusion, demonstrating the modular use of Rac GEFs in the control of distinct Rac-mediated events. We report that TIAM-1 functions mechanistically as a Rac-specific GEF and that TIAM-1 controls Rac GTPases in lamellipodia and filopodia formation. The roles of Rac and CDC-42 GTPases in lamellipodia and filopodia formation have been documented extensively, yet it is unclear how these molecules interact in this process. Here we show that TIAM-1 acts downstream of the CDC-42 GTPase, and might link CDC-42 and Rac signaling in a linear pathway in lamellipodia and filopodia formation downstream of UNC-40/DCC. Sif and Tiam1 have been previously implicated in axon development [33], [34]. We present evidence that TIAM-1 acts in the UNC-40/DCC pathway in axon guidance. Together, these data lead us to speculate that TIAM-1 controls growth cone lamellipodia and filopodia formation during axon guidance downstream of the UNC-40/DCC guidance receptor.

## Results

### C11D9.1 and T21E12.2 encode a GEF similar to mammalian Tiam1 and Drosophila Still Life

The *C11D9.1* gene is predicted to encode a molecule with DH and Pleckstrin homology (PH) domains most similar to mammalian T-cell lymphoma Invasion and Metastasis Factor 1 (Tiam1) and Drosophila Still Life (Sif), but lacks an apparent EVH1 domain and PDZ domain found in Tiam1 and Sif [33], [35]. Three cDNAs representing *C11D9.1* were obtained and sequenced (courtesy of Yuji Kohara). One of the cDNAs contained sequences complementary not only to *C11D9.1*, but also to the predicted upstream gene *T21E12.2* (Figures S1 and S2), indicating that *C11D9.1* and *T21E12.2* are a single gene.

A search of the additional predicted sequence encoded by *T21E12.2* using BLASTP and CCD [36], [37] did not identify regions similar to the EVH1 domain and PDZ domain of Tiam1 and Sif. However, two regions in the *C. elegans* molecule were conserved in the related nematodes *Ascaris Suum* and *Brugia malayi*, and these regions from *A. suum* and *B. Malayi* were recognized by BLASTP as being similar to the EVH1 and PDZ domain regions of Tiam1 and Sif (Figure S3). While these might not represent *bona fide* EVH1 and PDZ domains, this evolutionary comparison revealed regions in T21E12.2/C11D9.1 similar to the EVH1 domains and PDZ domains of Tiam1 and Sif. Mammalian Tiam1 contains an N-terminal myristoylation site (Myr) [38], [39], and a potential Myr site is found at the N-terminus of the predicted TIAM-1 molecule (Figure S2). We refer to the *T21E12.2/C11D9.1* locus collectively as *tiam-1*.

The predicted molecules encoded by the *tiam-1* cDNAs are shown in Figure S1. In other organisms, the DH-PH domain of Tiam1/Sif is a Rac-specific GEF [34], [40]. Both alleles of *tiam-1* used here (*ok772* and *tm1556*) remove at least part of the DH domain (Figures S1 and S2), and are predicted to cause frame shifts. Thus, *ok772* and *tm1556* are likely to be strong loss of function mutations.

### TIAM-1 interacts with Rac GTPases in axon pathfinding

**Figure 1:**
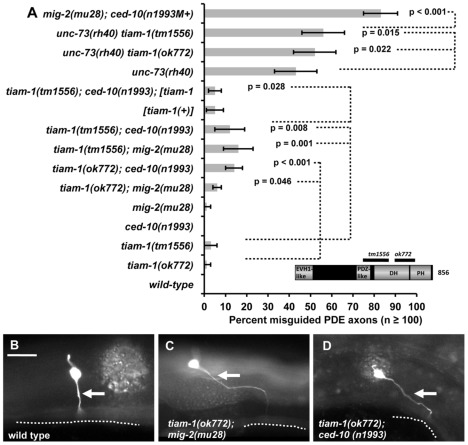
TIAM-1 acts with the Rac GTPases in axon pathfinding. A) A graph representing PDE axon pathfinding defects (X axis) in different genotypes (Y axis). At least 100 PDE neurons were scored, and p value significance was determined by Fisher's exact analysis. [tiam-1(+)] indicates a transgene that harbors a wild-type genomic copy of the *tiam-1* locus on fosmid clone WRM0633ch01. The error bars represent 2× standard error of the proportion in each direction. M+ indicates that the genotype had wild-type maternal gene function. The inset is a model of the predicted TIAM-1 isoform A molecule (856 amino acid residues) showing the regions of the molecule missing in two deletion alleles. Unless otherwise noted, all backgrounds are wild-type. p value significance as determined by Fisher Exact Analysis is shown. Dashed lines indicate comparisons between genotypes not marked with a p value to those marked with p values. (B–D) Fluorescent micrographs of *osm-6::gfp* in the PDE neurons of young adults. In all micrographs, dorsal is up and anterior is to the left. The arrows point to axons, which are misguided in mutants in (B) and (D). The ventral nerve cord is represented by a dashed line. The scale bar in (B) represents 5 µm for (B–D).

The *C. elegans* Rac-like GTPases MIG-2/RhoG and CED-10/Rac1 act redundantly in axon pathfinding [28]. Loss-of-function mutations in either *mig-2* or *ced-10* alone display few defects in pathfinding of the PDE axons (1% and 0%, respectively), but when in a double mutant combination (*mig-2(mu28); ced-10(n1993M+)*; M+ indicates that the genotype has wild-type maternally-supplied gene function), most PDE neurons were affected (84%, Figure 1A and [41]). UNC-73/Trio is a well characterized GEF for MIG-2/RhoG and CED-10/Rac1 in axon pathfinding [30]. A mutation that specifically abolishes the Rac GEF activity of UNC-73 (*rh40*, a Serine-1216-Phenylalanine (S1216F) substitution in the Rac-specific DH1 domain) [30] displayed severe axon pathfinding defects (42%, Figure 1A), but not as strong as the double mutant of *mig-2(mu28); ced-10(n1993M+)* (p<0.001). *mig-2(mu28); unc-73(rh40)* and *ced-10(n1993); unc-73(rh40)* doubles display pathfinding defects equivalent to *mig-2(mu28); ced-10(n1993M+)* [41]. Thus, another GEF might regulate Rac activity in axon pathfinding.

The *C. elegans* genome encodes 19 predicted DH-containing GEFs including UNC-73/Trio (Wormbase, and data not shown). We screened mutant alleles or RNAi of the other 18 DH-GEFs alone or in combination with *ced-10(n1993)* and *mig-2(mu28)* for PDE axon pathfinding defects (EAL, unpublished). The two deletion alleles of *tiam-1* described above, *ok772* and *tm1556*, caused low-penetrance defects in PDE axon pathfinding (1% and 3%, respectively) (Figure 1A). In double mutant combination with *mig-2(mu28)* and *ced-10(n1993)*, these defects were synergistically enhanced (Figure 1A–1C). For example, *tiam-1(tm1556); mig-2(mu28)* displayed 15% misrouted PDEs (p = 0.001), and *tiam-1(tm1556); ced-10(n1993)* 12% (p = 0.008). *tiam-1* mutations slightly but significantly enhanced *unc-73(rh40)* (Figure 1A; p = 0.022 and 0.015), indicating that TIAM-1 and UNC-73 might act in parallel in PDE axon guidance. Misguided axons often grew distances that were greater than the wild-type PDE axon, suggesting a defect in guidance rather than a general outgrowth defect. The fosmid clone WRM0633ch01 (Source Bioscience) carrying a wild-type copy of the *tiam-1* gene rescued the PDE axon pathfinding defects of *tiam-1(tm1556); ced-10(n1993)* (12% to 5%, p = 0.028) (Figure 1A). We noted that *tiam-1(+)* expression in a wild-type background caused a low level of PDE axon pathfinding defects (5%; Figure 1A), equivalent to the level in *tiam-1(tm1556); ced-10(n1993)* rescued animals. Thus, TIAM-1 transgenic expression caused defects in axon pathfinding.

The phenotype of *tiam-1(ok772); mig-2(mu28)* was significantly weaker than that of *tiam-1(tm1556); mig-2(mu28)* (p = 0.02) and other *tiam-1* doubles with *mig-2* and *ced-10* (Figure 1A). This might indicate that *ok772* retains some gene function and is a hypomorph. Alternatively, this might be the effect of genetic background. In any case, enhancement in *tiam-1(ok772); mig-2(mu28)* is still significant compared to *tiam-1(ok772)* alone (p = 0.046).

*tiam-1* mutants alone also displayed weak defects in other neurons. In the AVM neurons, *tiam-1(ok772)* displayed ventral axon pathfinding defects and 6% ectopic neurite formation, defects not seen in wild-type (Figure S4 and as discussed later). *tiam-1(ok772)* and *tiam-1(tm1556)* also displayed weak defects in VD/DD commissural motor neuron pathfinding (0.69% and 1.62%, respectively; p = 0.0051 for *tiam-1(tm1556)* but not significant for *tiam-1(ok772)*) (Figure S4).

These studies indicate that TIAM-1 displays a genetic interaction with the Rac GTPases CED-10 and MIG-2 and the UNC-73/Trio GEF in PDE axon pathfinding. That *tiam-1* enhances *mig-2* and *ced-10* could indicate that TIAM-1 acts in parallel to these GTPases or that TIAM-1 acts in both of the redundant MIG-2 and CED-10 pathways in pathfinding. Studies below indicate that TIAM-1 acts with both MIG-2 and CED-10. However, the *mig-2; ced-10* double mutant phenotype is much stronger than *tiam-1*, suggesting that another molecule might regulate MIG-2 and CED-1 in parallel to TIAM-1, possibly another GEF.

### A *tiam-1promoter::cfp* transgene is expressed in neurons

**Figure 2:**
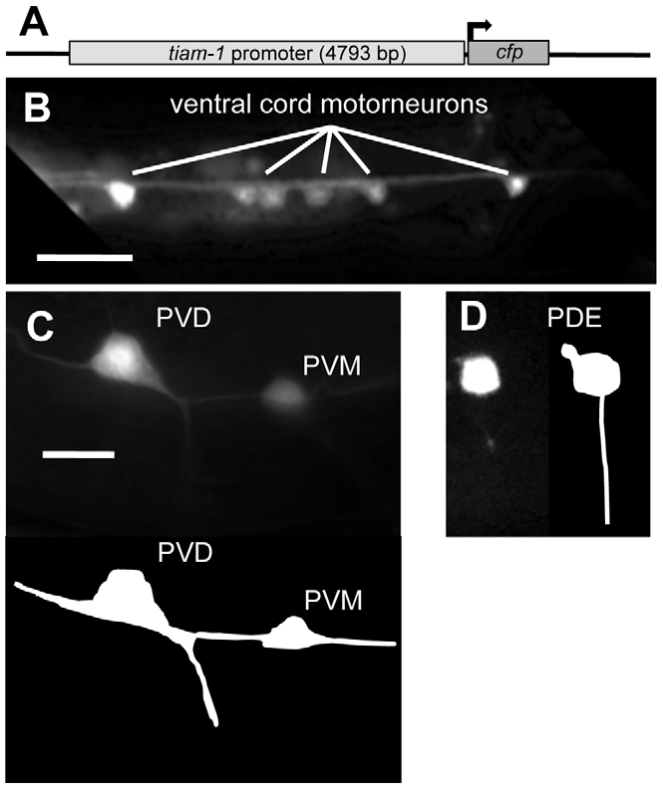
The *tiam-1* promoter drives expression in the nervous system. A) A diagram of the *tiam-1 promoter::cfp* transgene. The *tiam-1* promoter included 4793 bp upstream of the *tiam-1* A cDNA initiator codon. (B–D) Fluorescent micrographs of young adults harboring *tiam-1p::cfp*. In all micrographs, dorsal is up and anterior is to the left. B) *tiam-1p::cfp* expression in ventral cord motor neurons (ventral aspect). C) Expression in the post-deirid ganglion neurons PVD and PVM (lateral aspect) The diagram below depicts the positions of the neurons in the micrograph. D) Expression in the PDE neuron (lateral aspect), with a depiction to the right. The scale bar in (B) represents 10 µm, and the scale bar in (C) represents 2 µm for (C–D).

To determine where and when the *tiam-1* gene might by expressed, we studied expression of a *tiam-1* transcriptional reporter transgene. We generated by PCR a 4793-bp region upstream of T21E12.2 (the entire upstream region to the next predicted gene, T21E12.3) (Figure 2A), and this putative *tiam-1* promoter region was placed upstream of the *cyan fluorescent protein* (*cfp*) coding region. Expression of the *tiam-1promoter::cfp* construct was primarily in neurons (Figure 2B–2D). Most if not all neurons expressed the construct, including neurons in the head and tail, the ventral cord commissural motorneurons, the mechanosensory neurons (ALMs, PLMs, AVM, PVM) and the CAN, PDE, and PVD neurons. *tiam-1promoter::cfp* expression in neurons began in embryos and lasted throughout adulthood. No obvious expression outside of the nervous system was noted. However, *tiam-1* might be endogenously expressed in other tissues, as some transcriptional elements might not be present in this transgene.

### TIAM-1 is a Rac1-specific GTP exchange factor

**Figure 3:**
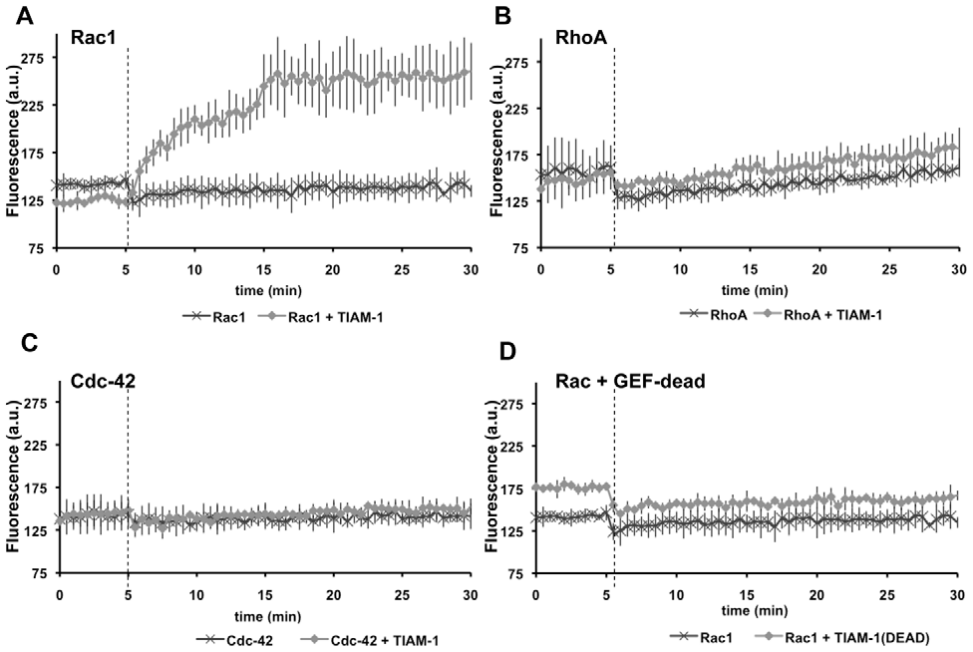
The TIAM-1 DHPH domain is a Rac1-specific GTP exchange factor. Graphs plotting arbitrary fluorescence units (A.U.) over time in a mant-GTP exchange assay (Cytoskeleton, Inc.) (see Methods). Dark, cross-hatched points represent exchange activity of the GTPase after the addition of vehicle without the DHPH domain of TIAM-1. Light, diamond-shaped points represent exchange after the addition of the DHPH domain of TIAM-1 in vehicle. The dashed vertical line represents the time of addition of vehicle or vehicle plus TIAM-1 DHPH. A) Exchange activity on human Rac1. B) Exchange activity on human RhoA. C) Exchange activity of human Cdc-42. D) Exchange activity on human Rac1 of a TIAM-1 DHPH domain with the T516F point mutation predicted to eliminate Rac GEF activity (TIAM-1(DEAD)). Error bars represent standard deviations of three independent experiments which each had consistent results.

*C. elegans* TIAM-1 is similar to the Rac GEFs Tiam1 and Sif. We tested if TIAM-1 from *C. elegans* acts as a GTP exchange factor *in vitro* using the mant-GTP RhoGEF exchange assay (Cytoskeleton, Inc; see Methods) (Figure 3). The DH-PH region of TIAM-1 was purified from bacterial expression and tested for exchange activity against human Rac1, Cdc42, and RhoA. In the absence of TIAM-1[DHPH], none of the GTPases incorporated mant-GTP (fluorescence did not change over 30 minutes). Addition of TIAM-1[DHPH] increased fluorescence with Rac1 (Figure 3A), but not Cdc-42 nor RhoA (Figure 3B–3C). This result suggests that TIAM-1[DHPH] has Rac1-specific GTP exchange activity.

The *unc-73(rh40)* S1216F missense mutation in the Rac-specific DH GEF domain of UNC-73 specifically abolishes Rac GEF activity [30]. We made an analogous mutation in TIAM-1[DHPH] (threonine 516 changed to phenylalanine (T516F) relative to the A isoform of TIAM-1 (Figure S2)). TIAM-1[DHPH] with the T516F mutation (TIAM-1(DEAD)) no longer showed exchange activity to Rac1 (Figure 3D), suggesting the T516F mutation abolished Rac1 GEF activity of TIAM-1[DHPH], similar to the *unc-73(rh40)* mutation S1216F. This mutation also abolished the ability of TIAM-1[DHPH] to induce ectopic protrusions in neurons as described below.

### The TIAM-1 and UNC-73 Rac GEF domains induce ectopic protrusion from neurons

**Figure 4:**
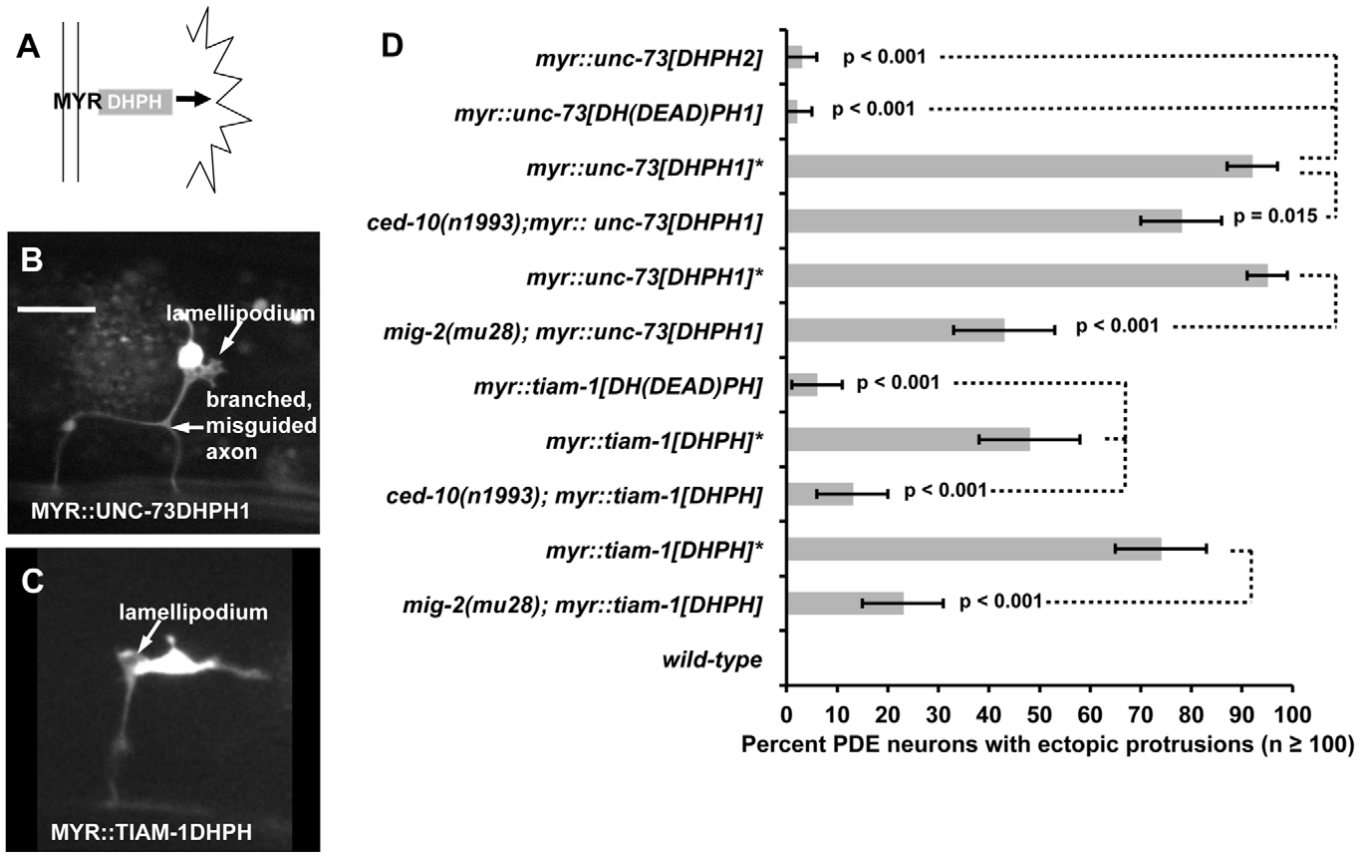
Ectopic protrusions induced by overactive TIAM-1 and UNC-73 were suppressed by loss-of-function of Rac GTPases. (A) Diagram of membrane-tethered, Rac-specific MYR::DHPH domains of UNC-73 and TIAM-1 inducing ectopic protrusions. (B–C) Fluorescent micrographs of PDE neurons with ectopic lamellipodial and filopodial protrusions (arrows) induced by MYR::DHPH constructs. Fluorescence is from soluble GFP expressed from an *osm-6::gfp* co-injection marker for cell shape. Compare to the wild-type PDE neurons in Figure 1. In all micrographs, dorsal is up and anterior is to the left. The scale bar in (B) represents 5 µm for (B–C). D) A graph plotting percentage of ectopic lamellipodial protrusions from the PDE neurons of young adult animals harboring transgenes driving the expression of MYR::DHPH domains of UNC-73 and TIAM-1. MYR::TIAM-1[DHPH] contains the TIAM-1 DHPH region (*lqIs165*). MYR::UNC-73[DHPH1] (*lqIs123*) contains the Rac specific GEF domain of UNC-73. A second DH GEF domain of UNC-73 is Rho specific (MYR::UNC-73[DHPH2]) (*lqIs125*). UNC-73[DH(DEAD)PH1] (*lqIs134*) and TIAM-1[DH(DEAD)PH] (*lqIs193*) are transgenes that harbor the point mutations that eliminate Rac GEF activity of the DH domains of UNC-73 DH1 and TIAM-1 (S1216F and T516F, respectively). Unless otherwise noted, all backgrounds are wild-type. At least 100 PDE neurons for each genotype were scored, and p value significance was determined using Fisher's exact analysis. Error bars represent 2× standard error of the proportion in both directions. The asterisk “*” indicates transgenes that have been crossed away from the *ced-10* or *mig-2* mutations and re-scored in a wild-type background. Dashed lines indicate comparisons between genotypes not marked with a p value to those marked with p values.

To test the roles of TIAM-1 and UNC-73 as potential Rac GEFs *in vivo*, we constructed transgenes that express constitutively-activated forms of the TIAM-1[DHPH] and UNC-73[DHPH1] Rac GEF domains in the PDE neurons. We based these experiments on previous results with Drosophila Trio (UNC-73 in *C. elegans*), in which a myristoylation sequence was added to the Rac-specific DHPH domain of Trio (Figure 4A). This construct produced a DHPH domain that was localized to cellular membranes via myristoylation [42]. Flies expressing this construct had retinal axon pathfinding defects due to unregulated activity of the DHPH domain. Mammalian Tiam1 has an N-terminal myristoylation site and is normally localized to membranes [38], [39], and TIAM-1 in *C. elegans* has a potential N-terminal myristoylation site (Figure S2). Furthermore, an N-terminal truncation of mammalian Tiam1, which does not affect the DHPH domains, activates Rac more strongly than full length Tiam1 [31], [43], suggesting that a deleted portion of Tiam1 might normally inhibit GEF activity of the molecule.

Based on these results, we created transgenes consisting of the TIAM-1 DHPH domain and both UNC-73 DHPH domains (Figure 4A) fused in frame to an N-terminal myristoylation sequence and to a C-terminal GFP (see Methods). Expression of these molecules in the PDE neurons was driven by the *osm-6* promoter and was confirmed by DHPH::GFP fluorescence in the PDE and other *osm-6*-expressing neurons (data not shown). The MYR::DHPH::GFP fusion proteins displayed a punctate, peripheral accumulation pattern consistent with localization to cellular membranes as seen in other MYR::GFP fusion proteins [44].

In a wild-type background, the Rac-specific MYR::UNC-73[DHPH1] induced ectopic protrusions that emanated from the cell body and axons of PDEs (Figure 4B). MYR::TIAM-1[DHPH] also promoted ectopic lamellipodial and filopodial protrusions (Figure 4C). Axon branching and defects in pathfinding were also observed in each case (Figure 4B and 4C). We generated multiple independent extrachromosomal transgenic lines with each construct, and in every case saw robust lamellipodial protrusions (Figure S5). In Drosophila, the Rho GEF domain of Trio did not cause retinal axon pathfinding defects [42]. We found that the Rho-specific MYR::UNC-73[DHPH2] of UNC-73 caused a low level of ectopic PDE protrusions in multiple transgenic extrachromosomal lines generated (Figure S5 and Figure 4D), significantly lower than the UNC-73 Rac-specific domain and MYR::TIAM-1DHPH (p≤0.001), consistent with the Drosophila studies.

To test the requirement of Rac GEF activity in ectopic protrusion, we introduced point mutations into the transgenes that abolish the Rac GEF activities of UNC-73 and TIAM-1 (the *unc-73(rh40) S1216F* mutation [30] and the T516F mutation in TIAM-1 used in Figure 3). The “GEF-dead” constructs were expressed as determined by GFP fluorescence. Multiple transgenic extrachromosomal lines were produced for each, and none promoted high levels of ectopic lamellipodial protrusions (Figure S5 and Figure 4D) (p≤0.001 compared to non-mutant transgenes). These results suggest that Rac GEF activity was required for robust ectopic lamellipodial and filopodial induction. These results also indicate that the MYR::[DHPH] transgenes likely produced constitutively activated GEF molecules rather than dominant negative molecules. We assayed the effects of MYR::TIAM-1[DHPH] in the *tiam-1(tm1556)* background and observed ectopic protrusions similar to the wild-type background (data not shown), consistent with the idea that MYR::TIAM-1DHPH represents a constitutively-activated rather than a dominant negative molecule.

### CED-10/Rac and MIG-2/RhoG are required for ectopic protrusions induced by activated TIAM-1 and UNC-73 GEF domains

To determine if Rac function was required for the effects of MYR::[DHPH] constructs, we crossed loss-of-function *mig-2* and *ced-10* with these overactive MYR::[DHPH] constructs to test for suppression of the ectopic protrusion phenotype. For these studies, we created integrated lines of the extrachromosomal transgenes described in Figure S5 (see Methods). In these experiments, we outcrossed the integrated transgene away from the loss-of-function mutation and re-scored the transgene to ensure that it retained the ability to induce ectopic protrusions (asterisks in Figure 4D). Both *mig-2(mu28)* and *ced-10(n1993)* significantly suppressed the ectopic projections caused by MYR::TIAM-1[DHPH] and MYR::UNC-73[DHPH1] (Figure 4D). *mig-2(mu28)* suppressed the ectopic lamellipodia and filopodia caused by MYR::UNC-73[DHPH1] (96% to 44%, p<0.001) and MYR::TIAM-1[DHPH] (76% to 23%, p<0.001). *ced-10(n1993)* suppressed MYR::TIAM-1[DHPH] strongly (48% to 13%, p<0.001) and MYR::UNC-73[DHPH1] more weakly but significantly (93% to 79%, p = 0.015). The same integrated MYR::TIAM-1[DHPH] transgene (*lqIs165*) was used in both the *mig-2* and *ced-10* double mutants, yet upon outcrossing, the transgene showed significantly different effects (p<0.01). We do not know the nature of this variability, but it might be due to effects of different genetic backgrounds after outcrossing. Regardless, in both cases the effects increased significantly upon outcrossing from *ced-10* and *mig-2*. We also scored integrated lines of the GEF dead constructs and the Rho-specific UNC-73 DHPH2 domain (Figure 4D), and found significantly reduced protrusions, consistent with results with extrachromosomal transgenes in Figure S5. These data indicate that unregulated Rac GEF activity of UNC-73 and TIAM-1 in the PDE neurons led to ectopic lamellipodia and filopodia that were dependent on CED-10/Rac1 and MIG-2/RhoG function. These results are consistent with the GTP exchange assays in Figure 3 and indicate that TIAM-1 interacts with CED-10/Rac1 and MIG-2/RhoG *in vivo*.

### TIAM-1 and Rac GTPases co-localize in *C. elegans* neurons

**Figure 5:**
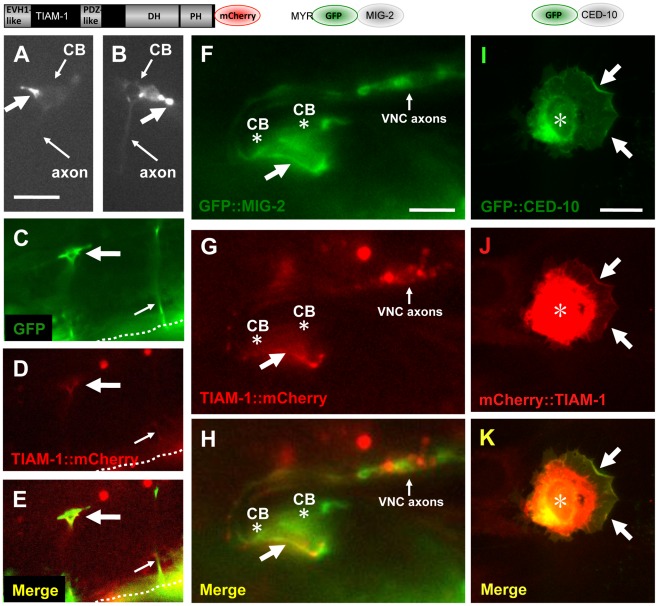
TIAM-1::mCherry is present in ectopic protrusions and growth cones and co-localizes with GFP::MIG-2 and GFP::CED-10. The diagrams above the micrographs represent the fusion proteins produced by the transgenes used: TIAM-1 C-terminally tagged with mCherry; MIG-2 N-terminally tagged with GFP after the predicted myristoylation site; and CED-10 N-terminally tagged with GFP. For the NIH 3T3 studies (I–K), an N-terminally-tagged TIAM-1 was assayed. Panels are fluorescent micrographs of animals harboring transgenes that express TIAM-1::mCherry, GFP, or GFP::MIG-2. In (A–H), dorsal is up and anterior is to the left. (A and B) Micrographs of PDE neurons of animals carrying an *osm-6p::tiam-1::mCherry* transgene expressed in the PDE neurons. TIAM-1::mCherry accumulated in ectopic protrusions that occurred at a low frequency (large arrows). Small arrows point to the cell bodies (CB) and the axons, which are faint. (C–E) A VD growth cone (large arrow) in an early L2 animal 18–20 hours post-hatching. The growth cone was visualized using cytoplasmic *unc-25p::gfp*, and also showed TIAM-1::mCherry accumulation driven from the *unc-25* promoter. The dashed line indicates the ventral nerve cord, and the small arrow points to a commissural motor axon that has completed commissural extension. The scale bar in (A) represents 5 µm for (A–E). (F–H) TIAM-1::mCherry and GFP::MIG-2 colocalized in ectopic protrusions. A ventral aspect of the ventral nerve cord is shown. (F) *unc-25p::gfp::mig-2* expression in an ectopic protrusion from VD or DD motor neurons (arrow). Cell bodies are indicated (CB, asterisk), as is the ventral cord neuropil (VNC axons). (G) *unc-25p::tiam-1::mCherry* expression in the ectopic protrusion of the same VD/DD neuron. (H) Co-localization of GFP::MIG-2 and TIAM-1::mCherry in the ectopic protrusion from the VD/DD neuron. The scale bar in (F) represents 3 µm for (F–H). (I–K) CED-10::GFP and TIAM-1::mCherry expression in NIH 3T3 cells. (I) GFP::CED-10 accumulated in membranous regions around the nucleus (asterisk), as well as at the periphery of lamellipodial ruffles induced by GFP::CED-10 (arrows). (J–K) TIAM-1::mCherry co-localized with GFP::CED-10 at the periphery of CED-10::GFP-induced lamellipodial ruffles (arrows). mCherry::TIAM-1 also accumulated to a perinuclear region that was more widespread than GFP::CED-10 accumulation (asterisk). The scale bar in (I) represents 10 µm for (I–K).

**Figure 6:**
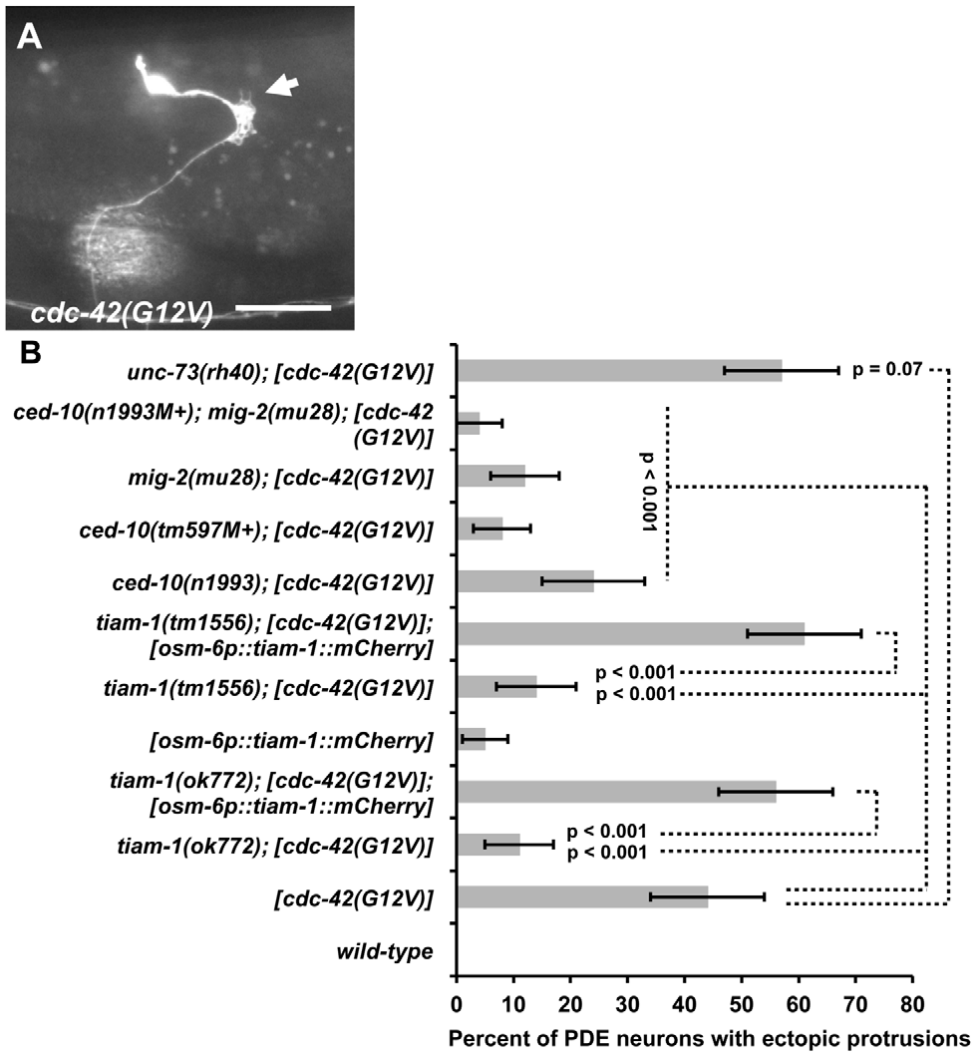
TIAM-1 acts cell autonomously downstream of CDC-42. (A) Fluorescent micrograph of a PDE neuron from an animal with an integrated *cdc-42(G12V)* transgene expressed with the *osm-6* promoter. An *osm-6::gfp* marker transgene was included to label the PDE cell body and processes. Dorsal is up and anterior is to the left. An arrow points to an ectopic lamellipodial protrusion. The scale bar in represents 5 µm. (B) A graph charting the percentage of PDE axons with ectopic lamellipodial and filopodial protrusions (X axis) in different genotypes (Y axis). Unless otherwise noted, all backgrounds are wild type. M+ indicates that the genotype had wild-type maternal gene function. *[cdc-42(G12V)]* indicates a transgene that harbors activated *cdc-42(G12V)* driven by the *osm-6* promoter in the PDEs. *[osm-6p::tiam-1(+)::mCherry]* indicates a transgene that harbors the corrected *tiam-1* cDNA yk730h9 fused in frame to *mCherry* and driven by the *osm-6* promoter in the PDE neurons (see Methods and Figure S2). At least 100 PDE neurons were scored for each genotype, and p value significance was determined using Fisher's Exact analysis. Error bars represent 2× standard error of the proportion in both directions. Dashed lines indicate comparisons between genotypes not marked with a p value to those marked with p values.

To determine the subcellular localization of TIAM-1 in neurons, we fused the longest isoform A *tiam-1* cDNA (yk730h9 in Figure S1 and S2) with *mCherry* at the C-terminus, and placed this construct under the control of the *unc-25* promoter (for VD/DD motor neurons) and the *osm-6* promoter (for PDE expression). The yk730h9 cDNA contained two errors relative to the reference gene models that led to frame shifts. These were repaired before proceeding (see Methods and Figure S2). This construct likely produced a functional TIAM-1::mCherry molecule, because it was required for ectopic protrusion induced by activated CDC-42(G12V) in the PDE neurons (see below and Figure 6), and it induced ectopic protrusions on its own (Figure 5A and 5B).

In the PDE and VD/DD neurons, TIAM-1::mCherry localized to puncta primarily at the periphery of the cell bodies, consistent with plasma membrane localization, as well as in the axons (Figure S6). Consistent with this observation, mammalian Tiam1 associates with membranes [38]. We noted that full-length TIAM-1::mCherry produced a low percentage (5%) of PDE and VD/DD neurons with ectopic neurites and protrusions (Figure 5A and 5B), similar to the TIAM-1 MYR::DHPH construct, suggesting that this molecule is active and drives ectopic protrusions. This is also consistent with the low level of PDE axon pathfinding defects observed upon *tiam-1(+)* expression in Figure 1A. TIAM-1::mCherry accumulated strongly in these ectopic protrusions (Figure 5A and 5B). TIAM-1::mCherry also accumulated in the growth cones of developing VD axons (Figure 5C–5E), consistent with a role of TIAM-1 in the growth cone during outgrowth.

We constructed *mig-2/RhoG* and *ced-10/Rac1* transgenes tagged with *gfp* driven by the *unc-25* promoter. MIG-2 contains an N-terminal myristoylation site and a C-terminal CAAX prenylation site. We placed *gfp* immediately after the Myr sequence in frame with *mig-2*, such that both myristoylated and prenylated forms of MIG-2 could be tagged with GFP. CED-10 was N-terminally tagged with GFP. GFP::CED-10 and GFP::MIG-2 accumulated at the cell periphery as previously described [28], [45] (data not shown). GFP::MIG-2 and TIAM-1::mCherry co-localized to the periphery of the ectopic protrusions of VD/DD neurons induced by expression of these molecules (Figure 5F–5H). GFP::CED-10 also co-localized with TIAM-1::mCherry (data not shown). Similar to TIAM-1::mCherry, GFP::CED-10 and GFP::MIG-2 were also found in the VD growth cones (data not shown). While TIAM-1::mCherry and GFP::MIG-2 and GFP::CED-10 showed co-localization, it was not completely overlapping co-localization (compare GFP in Figure 5F to mCherry in Figure 5G). This suggests that TIAM-1 and the GTPases MIG-2 and CED-10 might interact in specific regions in the cell (e.g. ectopic protrusions at the cell periphery).

### TIAM-1::mCherry and GFP::CED-10 co-localized to the plasma membrane in NIH-3T3 cultured mammalian fibroblasts

We analyzed the localization of N-terminally tagged mCherry::TIAM-1 and GFP::CED-10 in cultured NIH-3T3 fibroblasts. As expected, expression of GFP::CED-10 in fibroblasts caused marginal lamellipodial ruffling, a phenotype canonically associated with expression of vertebrate Rac1 in these cells (Figure 5I and Figure S7). GFP::CED-10 localized to intracellular regions around the nucleus that might represent membranous organelles, as well as to the plasma membrane, particularly in lamellipodial regions (Figure 5I and Figure S7A). Expression of mCherry::TIAM-1 did not affect cell shape as did GFP::CED-10, but did accumulate at the cell margins and intracellular membranous organelles (Figure S7B). These patterns are likely to represent CED-10 and TIAM-1 specific localization, as mCherry and GFP expression alone did not show these patterns (Figure S7C and S7D).

When expressed together, mCherry::TIAM-1 and GFP::CED-10 co-localized to the lamellipodial regions of the plasma membrane induced by GFP::CED-10 (Figure 5I–5K). Both molecules also localized to a perinuclear region, but overlap was not complete (TIAM-1::mCherry was more widely distributed in these perinuclear regions than was GFP::CED-10; compare Figure 5I and 5J). Similar to the results in *C. elegans*, these data indicate that TIAM-1 and CED-10/Rac1 might interact in specific regions of the cell. In 3T3 cells, they co-localize where CED-10/Rac1 induces lamellipodial ruffles at the cell periphery (Figure 5K). Possibly, TIAM-1 and the GTPases MIG-2 and CED-10 co-localize only where TIAM-1 is activating them (i.e. acting as a Rac GEF).

### TIAM-1 acts cell-autonomously downstream of CDC-42

Evidence from cell and neuronal culture studies [46], [47] suggests mammalian Tiam1 acts downstream of Cdc-42 in the activation of Rac1 in axon development. We tested if TIAM-1 acts downstream of CDC-42 in *C. elegans*. A glycine-12-valine substitution in the GTPase binding pocket of small GTPases has been shown to favor the GTP-bound state of these molecules, constitutively activating them [41]. Previous studies have shown that Rac(G12V) activity (CED-10/Rac and MIG-2/RhoG) in *C. elegans* neurons induces ectopic lamellipodia and filopodia, similar to those described for MYR::TIAM-1(DHPH) (Figure 4) [41]. We constructed a CDC-42(G12V) transgene driven by the *osm-6* promoter in the PDE neurons. Transgenic animals harboring *cdc-42(G12V)* displayed ectopic lamellipodial and filopodial protrusions (44%) (Figure 6A), as well as disruption of proper axon pathfinding (50%; n = 100) in the PDE neurons, similar to Rac(G12V) and MYR::TIAM-1(DHPH). Thus, unregulated CDC-42 activity promotes lamellipodial and filopodial formation in neurons. We generated multiple *cdc-42(G12V)* transgenic lines, and each induced ectopic protrusions. We used one representative line for further studies.

To test if TIAM-1 was required for the effects of CDC-42(G12V), we assayed the effects of *cdc-42(G12V)* in *tiam-1(ok772)* and *tiam-1(tm1556)* mutants. In both cases, the ectopic lamellipodia and filopodia induced by CDC-42(G12V) were significantly suppressed (12% for *ok772* and 15% for *tm1556*, p<0.0001) (Figure 6B). These data suggest that TIAM-1 is required for the activity of CDC-42(G12V). In contrast, *unc-73(rh40)* did not suppress CDC-42(G12V) (47% ectopic lamellipodia and filopodia in *unc-73(rh40); cdc-42(G12V)* compared to 44% in *cdc-42(G12V)* alone; p = 0.07)). These data suggest that TIAM-1, but not UNC-73, is required for the effects of CDC-42(G12V).

*osm-6p::tiam-1::mCherry*, which produces full-length TIAM-1 tagged with mCherry in the PDE neurons but not surrounding cells, restored the effects of CDC-42(G12V) in *tiam-1(ok772)* and *tiam-1(tm1556)* mutants (Figure 6B) (p<0.001 in each case). These results indicate that TIAM-1 is required cell autonomously for the ectopic lamellipodia and filopodia induced by CDC-42(G12V) in the PDE neurons. A genomic fosmid containing *tiam-1* with its endogenous promoter also restored CDC-42(G12V)-induced ectopic lamellipodia and filopodia (Figure S8).

With *osm-6p::tiam-1::mCherry* rescue, the effects of CDC-42(G12V) were consistently enhanced compared to CDC-42(G12V) alone (p = 0.079 for ok772 rescue, and p = 0.014 for *tm1556* rescue). This was also the case with fosmid rescue (p = 0.106 for ok772 rescue, and p = 0.02 for *tm1556* rescue). Possibly, the excess TIAM-1 expressed from the transgene is activated by CDC-42(G12V), leading to increased defects. However, TIAM-1 expression alone causes ectopic protrusions (5%; Figure 6B), possibly explaining the increase.

**Figure 7:**
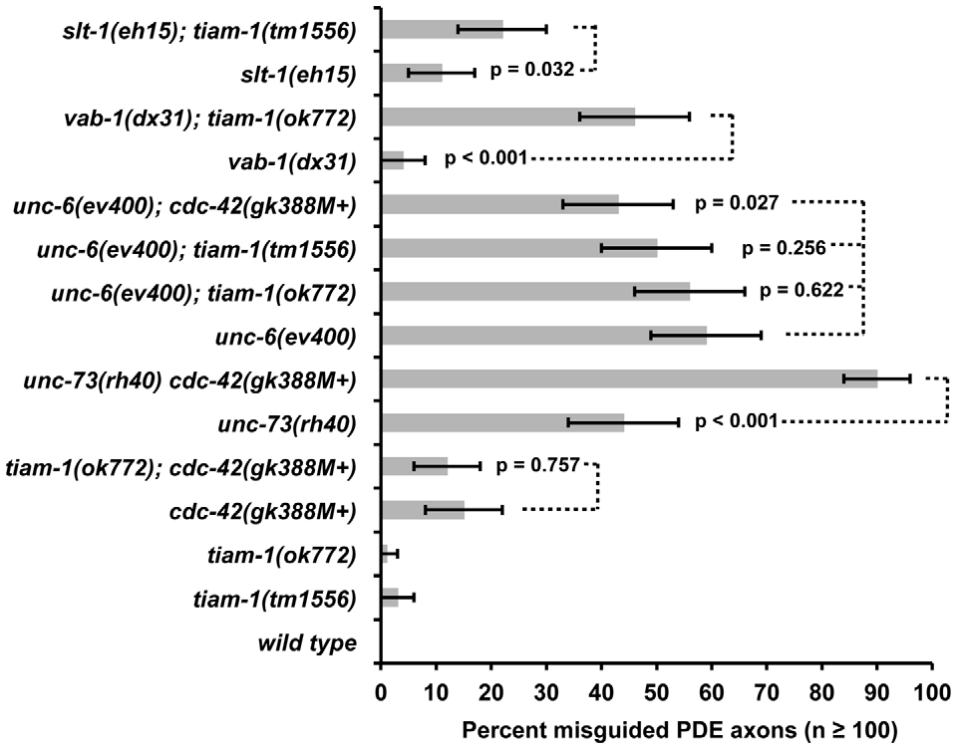
*tiam-1 and cdc-42* interactions with axon guidance mutations. A graph representing PDE axon pathfinding defects (X axis) in different genotypes (Y axis). At least 100 PDE neurons were scored, and p value significance was determined by Fisher's exact analysis. The error bars represent 2× standard error of the proportion in each direction. M+ indicates that the genotype had wild-type maternal gene function. Dashed lines indicate comparisons between genotypes not marked with a p value to those marked with p values.

These findings were corroborated with loss-of-function studies. The *cdc-42(gk388)* deletion removes the initiator codon and a large portion of the coding region and is likely a null allele (Wormbase). *cdc-42(gk388M+)* homozygotes, with wild-type maternal gene function, survived until late larval/early adulthood and were sterile. Alone, *cdc-42(gk388M+)* animals display 15% PDE axon pathfinding defects (Figure 7). *tiam-1(ok772); cdc-42(gk388M+)* animals showed no increase in pathfinding defects and resembled *cdc-42(gk388M+)* alone. However, *unc-73(rh40); cdc-42(gk388M+)* mutants displayed a synergistic increase in the number of PDE pathfinding defects (90%) compared to *cdc-42(gk388M+)* and *unc-73(rh40)* alone (p<0.001) (Figure 7). These loss-of function studies are consistent with the idea that TIAM-1 and CDC-42 act in the same pathway in PDE axon guidance, possibly in parallel to UNC-73/Trio.

### CED-10/Rac1 and MIG-2/RhoG are required for the effects of CDC-42(G12V)

Our results suggest that TIAM-1 acts downstream of CDC-42 in ectopic lamellipodial protrusion. Given that TIAM-1 can act as a Rac GEF, we expect that MIG-2/RhoG and CED-10/Rac1 might also be required for the effects of CDC-42(G12V). Indeed, *mig-2(mu28)* and *ced-10(n1993)* suppressed the effects of CDC-42(G12V) (Figure 6B; p<0.001). The *ced-10(tm597M+)* deletion allele also suppressed CDC-42(G12V) (p<0.001). Suppression by *ced-10(n1993)* was significantly weaker than that of *mig-2(mu28)* (24% compared to 12%; p = 0.022) and *ced-10(tm597M+)* (8%; p = 0.001). *ced-10(n1993)* is a mutation in the C-terminal prenylation sequence and is a hypomorph, consistent with weaker suppression [48]. The *ced-10(n1993M+); mig-2(mu28)* double mutant suppressed (4%, p<0.001) slightly better than *mig-2(mu28)* or *ced-10(tm597M+)* alone (p = 0.029 and 0.0194, respectively). These data indicate that CED-10/Rac1 and MIG-2/RhoG are required for the ectopic protrusions caused by CDC-42(G12V).

### TIAM-1 might act in the UNC-6/Netrin pathway in ventral axon guidance

We have shown evidence that the Rac GEF TIAM-1 regulates the Rac GTPases CED-10 and MIG-2 in response to CDC-42 signaling. We next sought to determine which dorsal-ventral axon guidance ligand/receptor pathway might be upstream of TIAM-1. Distinct parallel ventral guidance pathways control dorsal ventral guidance in *C. elegans*. UNC-6/Netrin is a secreted ligand expressed in the ventral side of the animal that attracts axons that express its receptor UNC-40/DCC [49]. SLT-1/Slit is expressed primarily in the dorsal side and is thought to act as a repellent for ventrally-guided axons that express its receptor SAX-3/Robo [50]. Ephrins have also been involved in directing proper axon pathfinding [51]. VAB-1/EphR is the only *C. elegans* Ephrin receptor [52] and has been shown to work in parallel with UNC-6/Netrin and SLT-1/Slit in axon ventral guidance [53].

We constructed double mutants of *tiam-1* with the loss-of-function alleles *unc-6(ev400), slt-1(eh15), and vab-1(dx31)*, and scored defects in PDE axon ventral guidance (Figure 7). *tiam-1(ok772)* significantly increased the defects of *vab-1(dx31)* (4% to 46%, p<0.001), and *tiam-1(tm1556)* enhanced *slt-1(eh15)* (11% to 22%, p = 0.032), suggesting that TIAM-1 might act in parallel to VAB-1/Eph and SLT-1/Slit. In contrast, neither *tiam-1(ok772)* nor *tiam-1(tm1556)* increased the amount of PDE pathfinding defects of *unc-6(ev400)* (from 59% to 56% and 50%; p = 0.622 and 0.256). *slt-1(eh15)* is a small deletion and is likely a null [50], an *vab-1(dx31)* is a complex rearrangement that deletes exons 1–4 and is also likely a null [52]. That *tiam-1* enhanced *slt-1* and *vab-1* but did not enhance *unc-6* is consistent with TIAM-1 acting in the UNC-6/Netrin pathway in parallel to VAB-1 and SLT-1 in PDE ventral guidance.

### TIAM-1 might act downstream of UNC-40/DCC

**Figure 8:**
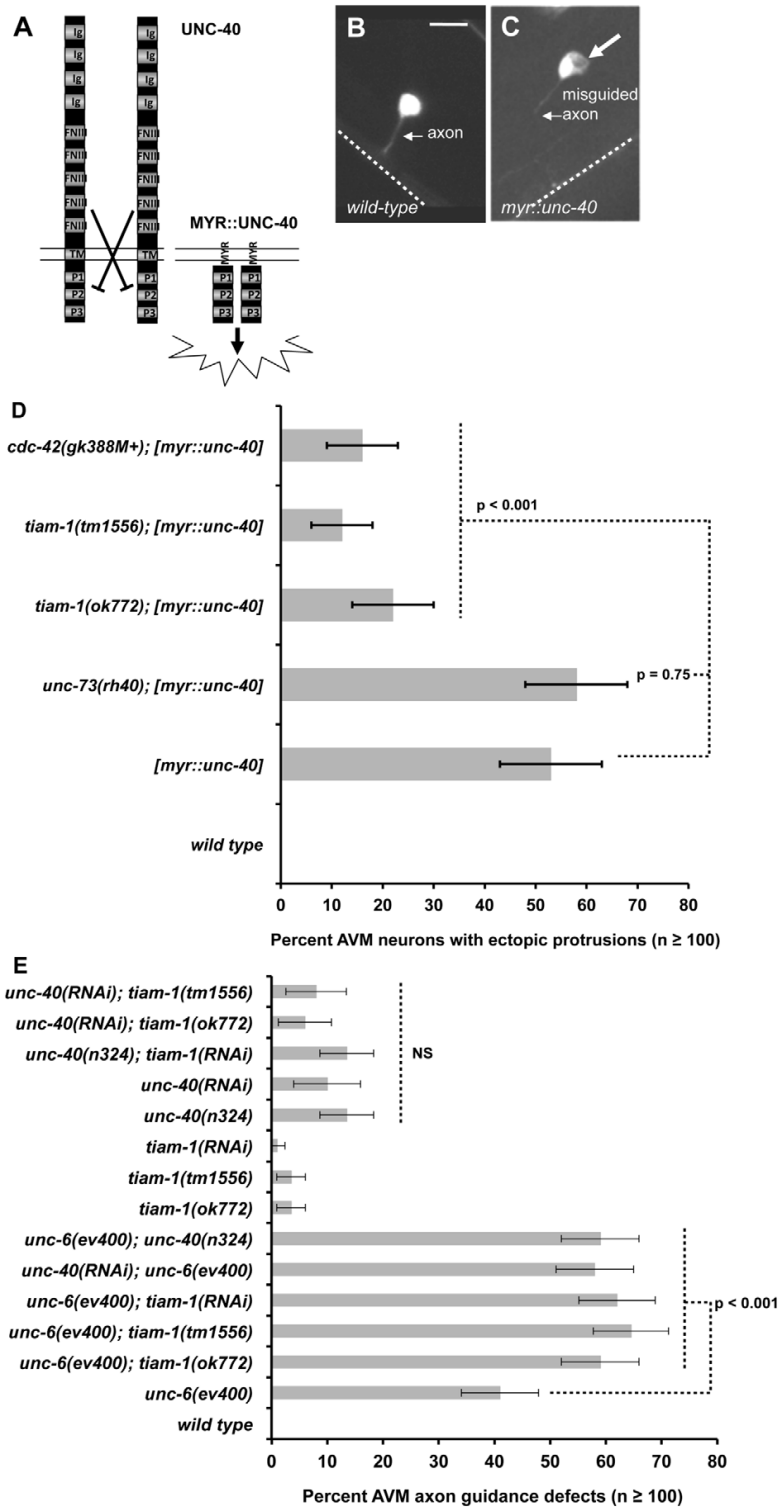
TIAM-1 acts with UNC-6/netrin and UNC-40/DCC in axon guidance and protrusion. (A) A diagram illustrating attractive axon guidance via the UNC-40/DCC receptor. In the absence of UNC-6/Netrin ligand, the extracellular domains of UNC-40 prevent dimerization of the cytoplasmic regions and activation of receptor signal transduction. A myristoylated version of the cytoplasmic domain lacking the transmembrane domain and extracellular domains is believed to constitutively dimerize and is constitutively active, resulting in ectopic protrusion from the AVM neuron. Ig = immunoglobulin domain; FNIII = fibronectin type II domain; TM = transmembrane domain; P1–P3 = conserved proline-rich domains; MYR = covalent myristoyl group. (B) A wild-type AVM neuron visualized with soluble GFP from a *mec-4::gfp* transgene, with an arrow pointing to the unbranched ventrally-directed axon. The dashed line indicates the ventral nerve cord. (C) An AVM neuron with expression of *unc-86p::myr::unc-40*, a transgene that drives a myristoylated version of the cytoplasmic domain of UNC-40 in AVM. Fluorescence is soluble GFP expressed from *mec-4::gfp* to highlight cell shape. A large arrow points to an ectopic lamellipodium, and a smaller arrow points to a misguided and branched AVM axon. In all micrographs, dorsal is up and anterior is to the left. The scale bar represents 5 µm for B and C. (D) A graph charting the percentage of AVM axons with ectopic lamellipodial protrusions (X axis) in different genotypes (Y axis). Unless otherwise noted, all backgrounds are wild type. *[myr::unc-40]* represents animals harboring a transgene that expresses the myristoylated UNC-40 cytoplasmic domain under the *mec-7* promoter (*mec-7p::myr::unc-40*). M+ indicates wild-type maternal gene function. Alone, none of the single mutants *tiam-1(ok772)*, *cdc-42(gk388M+)*, or *unc-73(rh40)* displayed ectopic protrusions. (E) A graph plotting the percentage of animals with AVM ventral axon guidance defects in different genotypes. Reported here are defects in AVM guidance that include failure of the axon tom reach the ventral nerve cord and axon wandering. If we just consider failure to reach the ventral nerve cord, similar significant results are found (*unc-40* and *tiam-1* enhance *unc-6(ev400)*, and *tiam-1* does not enhance *unc-40*). At least 100 AVM neurons were scored for each genotype, and p value significance was determined using Fisher's Exact analysis. Error bars represent 2× standard error of the proportion in both directions. Dashed lines indicate comparisons between genotypes not marked with a p value to those marked with p values. NS = not significantly different.

Previous studies indicated that the Rac GTPase CED-10 acts downstream of the UNC-6/Netrin receptor UNC-40/DCC in the AVM neuron [9]. A myristoylated form of the cytoplasmic tail of the Netrin receptor UNC-40 missing the extracellular and transmembrane domains induced axon defects and ectopic lamellipodia and filopodia in the AVM neurons due to constitutive activation of UNC-40 signaling (see diagram in Figure 8A) [9]. Loss of *ced-10/Rac1* function suppressed *myr::unc-40*, but loss of *unc-73/Trio* did not [9], suggesting a distinct GEF might act downstream of UNC-40.

We tested if TIAM-1 might act downstream of MYR::UNC-40 in AVM. When expressed in the PDE neurons, MYR::UNC-40 did not result in ectopic lamellipodial protrusions. We do not know why this is the case, but possibly the PDE neuron is less sensitive to the effects of MYR::UNC-40 than is AVM, or the *osm-6* promoter is not expressed at the correct time or level. We expressed MYR::UNC-40 in the AVMs using the *mec-7* promoter as previously described [9]. In our hands, MYR::UNC-40 caused 53% of AVMs to form ectopic lamellipodial protrusions (Figure 8B, 8C, and 8D). As expected, *unc-73(rh40)* did not suppress (Figure 8D; 58%), confirming the results described previously [9]. However, *tiam-1(ok772)* significantly suppressed the ectopic lamellipodia incidence in these neurons (22%, p<0.001). Furthermore, *cdc-42(gk388M+)* suppressed MYR::UNC-40 (16%, p<0.001), suggesting that CDC-42 is also downstream of UNC-40. Consistent with this idea, *cdc-42(gk388M+)* did not enhance *unc-6(ev400)* in PDE ventral axon guidance (Figure 7). In fact, *cdc-42(gk388M+)* slightly suppressed *unc-6(ev400)* (p = 0.027). The nature of this possible suppression is unclear, but it is possible that *cdc-42(gk388M+)* might also normally attenuate UNC-6/Netrin signaling. While *tiam-1* loss of function suppressed the ectopic protrusions driven by MYR::UNC-40, the axon guidance and branching defects were not suppressed significantly (data not shown), suggesting that MYR::UNC-40 perturbation of axon guidance and branching are not dependent upon TIAM-1 and might be due to effects distinct from ectopic lamellipodial protrusion.

We assayed AVM axon guidance in single and double loss-of-function mutants involving *unc-6*, *unc-40*, and *tiam-1* (Figure 8E). *unc-6(ev400)* and *unc-40(n324)* both displayed defective AVM ventral guidance as expected, but *unc-6(ev400)* was significantly stronger than *unc-40(n324)* (41% versus 14%; p<0.001). Both are predicted null mutations, suggesting that UNC-40 might not be the only receptor for UNC-6 in AVM ventral guidance. AVM defects in *unc-6(ev400)* mutants were scored previously by another group [54], and fewer defects were noted in this study (∼25% compared to 41% in our hands). We scored AVM guidance as defective if the axon failed to reach the ventral cord or if the axon wandered more than 45 degrees away from straight ventrally. When we consider only axons that failed to reach the ventral nerve cord, we see 27% defects in *unc-6(ev400)* (data not shown), similar to those previously described [54].

We found that *tiam-1(tm1556)*, *tiam-1(ok772)*, and *tiam-1(RNAi)* significantly enhanced the AVM ventral guidance defects of *unc-6(ev400)* (Figure 8E). This result is in contrast to the PDE, where *tiam-1* did not enhance *unc-6* (Figure 8D), suggesting cell-specific differences in TIAM-1 function with UNC-6. While *tiam-1* enhanced *unc-6*, we found that *tiam-1* did not enhance *unc-40*. For these studies we used RNAi, as *tiam-1* and *unc-40* are closely linked and we were unable to construct a double mutant. *unc-40(RNAi)* caused defects in AVM guidance similar to *unc-40(n324)* that were not enhanced by *tiam-1* mutations; and *tiam-1(RNAi)*, which enhanced *unc-6(ev400)*, did not enhance *unc-40(n324)* (Figure 8E). These data are consistent with TIAM-1 acting in the UNC-40/DCC pathway. In further support of this notion, we found that *unc-40(n324)* and *unc-40(RNAi)* enhanced *unc-6(ev400)* in a manner similar to *tiam-1*. Enhancement of *unc-6(ev400)* by *tiam-1* and *unc-40* was also significant when only failure to reach the ventral nerve cord was considered (data not shown). Previous results showed that *unc-40(e1430); unc-6(ev400)* doubles had only slightly increased AVM guidance defects (∼25% to ∼33%) [54]. We used *unc-40(n324)* and *unc-40(RNAi)* and the previous study used *unc-40(e1430)* [54], raising the possibility that the stronger enhancement of *unc-6(ev400)* that we report is due to different *unc-40* alleles used.

In sum, these genetic interactions suggest that UNC-6 and UNC-40 act partially in parallel and also suggest that UNC-6 might not be the only ligand for UNC-40/DCC in AVM ventral guidance. They are also consistent with TIAM-1 acting in the UNC-40 pathway in AVM ventral axon guidance. That *unc-40* has a more severe phenotype than *tiam-1* suggests that additional molecules might act in parallel to TIAM-1 downstream of UNC-40.

## Discussion

**Figure 9:**
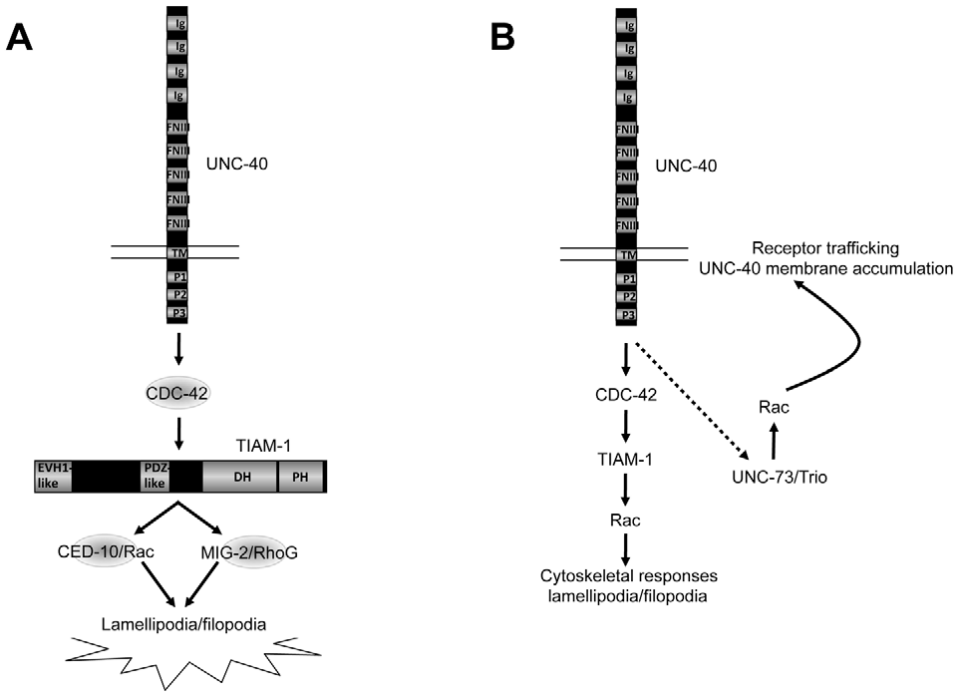
A model of the distinct roles of the Rac GEFs TIAM-1 and UNC-73/Trio in axon guidance. Arrows represent functional or genetic interactions between molecules and do not imply physical interactions. A) TIAM-1 controls protrusive events downstream of UNC-40/DCC. Results presented here suggest that TIAM-1 acts downstream of UNC-40/DCC and CDC-42, and upstream of the Rac GTPases CED-10 and MIG-2, in ectopic lamellipodial and filopodia formation and attractive axon guidance. B) TIAM-1 and UNC-73/Trio might regulate distinct aspects of Rac function in UNC-40/DCC-mediated axon guidance. TIAM-1 might regulate Rac GTPases downstream of UNC-40/DCC involving cytoskeletal protrusive events. UNC-73/Trio might control Rac GTPases upstream of UNC-40/DCC in trafficking of UNC-40/DCC to the membrane [55], [62]. The dashed arrow indicates a potential feedback loop where by UNC-40/DCC might control its own membrane trafficking via UNC-73. Ig = immunoglobulin I domain; FNIII = fibronectin type III domain; TM = transmembrane domain; P1–P3 = conserved proline-rich domains; EVH1 = EVH1 domain; PDZ = PDZ domain; DH = Dbl-homology domain; PH = Pleckstrin homology domain.

In recent years, attention has focused on how guidance receptors engage cytoplasmic signaling mechanisms to control growth cone outgrowth and turning during axon guidance. In this report we present evidence of a signaling pathway (Figure 9A) involving the previously uncharacterized *C. elegans* molecule TIAM-1, which acts specifically as a GEF for Rac GTPases and interacts with them in axon guidance and neuronal protrusion. We show that TIAM-1 regulates neuronal protrusion downstream of the CDC-42 GTPase and upstream of the two redundant Rac GTPases MIG-2/RhoG and CED-10/Rac1. Genetic interactions of these molecules suggest that that they act in a common pathway in axon pathfinding. Furthermore, we provide evidence that TIAM-1 acts with the UNC-6/Netrin receptor UNC-40/DCC in axon guidance and downstream of UNC-40/DCC in neuronal protrusion. Previous studies have implicated Rac GTPases and the Rac GEF Trio as players in Netrin signaling and interaction with the Netrin receptor DCC [55]–[57]. Here we present evidence that the Rac GEF TIAM-1, along with CDC-42, CED-10/Rac1, and MIG-2/RhoG, acts downstream of UNC-40/DCC in the formation of neuronal protrusions, which might reflect a role in protrusive activity in the growth cone during outgrowth.

### TIAM-1 links CDC-42 and Rac GTPases in neuronal protrusion downstream of UNC-40/DCC

GTP exchange assays indicated that TIAM-1 is a Rac-specific GEF (Figure 3). The activated Rac-specific MYR::DHPH domains of TIAM-1 and UNC-73 induced ectopic protrusions from PDE neurons that resembled lamellipodia and filopodia. Protrusions were dependent upon a functional DH GEF domain in these molecules as well as on the presence of MIG-2/RhoG and CED-10/Rac1. TIAM-1 was required for ectopic protrusions induced by CDC-42 and activated MYR::UNC-40; and CDC-42 was required for MYR::UNC-40-induced protrusions. Together, these data suggest a pathway of neuronal lamellipodial and filopodial protrusion wherein TIAM-1 links CDC-42 and Rac GTPase signaling downstream of UNC-40/DCC.

A caveat with the studies using DHPH Rac GEF domains of TIAM-1 and UNC-73 is that the GEF domains were not in the context of the full-length molecules, and thus these MYR::DHPH constructs might not reflect the normal cellular roles of these molecules in neurons. These studies do indicate, however, that TIAM-1 and UNC-73 have the ability to act as Rac GEFs *in vivo*, whether or not their normal role is in protrusion. However, we found that transgenic expression of full-length TIAM-1::mCherry also induced ectopic protrusions in the PDE neurons similar to but at a much lower level than the MYR::DHPH construct, suggesting that regulation of protrusion is a normal cellular role of TIAM-1. This is consistent with *tiam-1* suppression of protrusion caused by CDC-42(G12V) and MYR::UNC-40. In contrast, *unc-73* suppressed neither of these, suggesting that UNC-73/Trio might not normally regulate protrusive events downstream of UNC-40. We speculate that UNC-73 might control Rac activity upstream of guidance receptors to control their cell surface trafficking (Figure 9B and see below).

TIAM-1::mCherry co-localized with GFP::MIG-2 in ectopic protrusions from *C. elegans* neurons, and with GFP::CED-10 in lamellipodial ruffles induced by GFP::CED-10 in cultured NIH 3T3 fibroblasts. That these molecules did not co-localize in other regions of the cell suggests that TIAM-1 associates with CED-10/Rac1 and MIG-2/RhoG only where they are actively inducing protrusion.

TIAM-1 displayed genetic interactions with the Rac GTPases in PDE axon guidance. *tiam-1* mutations enhanced mutations in *mig-*2 and *ced-10*, which have redundant roles in axon guidance [15], [24], [28], [41]. Thus, TIAM-1 might act with both molecules. This idea is supported by gain-of-function studies showing that both MIG-2 and CED-10 are required for the ectopic protrusions induced by the activated MYR::DHPH domain of TIAM-1. TIAM-1 might act in parallel to another molecule, possibly another GEF, that regulates Rac GTPases in axon guidance, as the *mig-2; ced-10* double mutant phenotype is more severe than *tiam-1*.

### Genetic interactions suggest that TIAM-1 acts in the UNC-40/DCC axon guidance pathway

The UNC-6/Netrin ligand is expressed in ventral cells, and growth cones are attracted to or repelled from the ventral source [58]. A series of loss-of-function genetic interactions suggest that CDC-42, TIAM-1, MIG-2/RhoG, and CED-10/Rac1 act in the UNC-40/DCC axon guidance pathway. In PDE axon guidance (Figure 8D), *tiam-1* enhanced *slt-1/Slit* and *vab-1/EphR* but not *unc-6/Netrin.* In AVM axon guidance (Figure 8E), *tiam-1* enhanced *unc-6*, suggesting cell specific differences in the roles of these molecules in axon guidance. However, *tiam-1* did not enhance *unc-40* in AVM axon guidance, suggesting that TIAM-1 might act with UNC-40. Consistent with this idea, *unc-40* also enhanced *unc-6* in AVM guidance, similar to *tiam-1*. However, *tiam-1* mutations were less severe than *unc-40*, suggesting that other molecules act in parallel to TIAM-1 in UNC-40 signaling. Indeed, the TRIM molecule MADD-2 acts with UNC-6 and UNC-40 signaling in AVM ventral guidance, but is not required for the protrusive effects of MYR::UNC-40 [54]. We find that TIAM-1 is required for MYR::UNC-40 protrusive effects but not axon guidance defects caused by MYR::UNC-40, suggesting that distinct pathways might act downstream of UNC-40 to mediate distinct roles of UNC-40 in axon guidance (i.e. protrusive versus non-protrusive events).

These results have implications about UNC-6/Netrin signaling in axon guidance. First, *unc-6* AVM axon guidance defects were more severe than *unc-40*, suggesting that UNC-6 signals through other receptors in addition to UNC-40/DCC. Second, that *unc-40* enhances *unc-6* suggests that these molecules act at least in part in parallel, and suggest that UNC-6 might not be the only ligand for UNC-40/DCC in AVM ventral guidance. Possible candidates for such interactions might be the SLT-1/Slit ligand and its receptor SAX-3/Robo, which have been shown to functionally interact with Netrin and DCC [59]. In any case, genetic interactions suggest that TIAM-1 acts in the UNC-40 pathway in AVM axon guidance.

### The relationship of lamellipodial and filopodial protrusion to axon guidance

Our results demonstrate that TIAM-1 is required for axon guidance and that TIAM-1 regulates lamellipodial and filopodial protrusions in neurons. Growth cones, including those in *C. elegans*, display dynamic lamellipodial and filopodial protrusions [19], [60]. Our recent findings indicate that the Netrin receptors UNC-40/DCC and UNC-5 modulate growth cone lamellipodial and filopodial protrusion that correlates with attractive versus repulsive responses to UNC-6/Netrin (i.e. UNC-6/Netrin inhibits protrusion in repelled growth cones via UNC-5, and stimulates protrusion in attracted growth cones via UNC-40/DCC) [61]. We speculate that TIAM-1 normally stimulates growth cone lamellipodial and filopodial protrusion in response to UNC-40/DCC in UNC-6/Netrin attractive axon guidance (Figure 9). While it is possible that TIAM-1 controls axon guidance by a mechanism distinct from lamellipodia and filopodia formation, this model is consistent with our data presented here.

### Distinct roles of TIAM-1 and UNC-73/Trio in axon guidance

Our results and those of others [9] suggest that UNC-73/Trio acts in parallel to UNC-6/Netrin signaling in PDE axon guidance, and might not act downstream of UNC-40/DCC in AVM neuronal protrusion. In contrast to these results, Trio has been shown in multiple systems to act with Rac GTPases in Netrin signaling. In *C. elegans*, UNC-73/Trio and the kinesin-like VAB-8L molecule interact and control accumulation of the UNC-6/Netrin receptor UNC-40/DCC and the SLT-1/Slit receptor SAX-3/Robo to the cell surface (i.e. UNC-73/Trio acts upstream of UNC-40/DCC to control its accumulation) [55], [62]. In Drosophila, Trio and the Abelson kinase Abl interact with the Netrin receptor Frazzled (the fly equivalent of UNC-40 and DCC) to control guidance events involving Netrin [56]. Trio was required for the ectopic midline crossing of axons caused by a hybrid chimeric Frazzled-Robo receptor composed of the extracellular and transmembrane domains of Robo and the cytoplasmic domain of Frazzled. In vertebrates, Trio is required for Netrin mediated activation of Rac1 in neurons, and is required for neurite extension in response to Netrin [57].

How might our results fit with these known roles of Trio in Netrin signaling? Cell-specific and context-specific differences in Trio function downstream of guidance receptors are possible explanations. In other words, Trio might act with UNC-40/DCC in some neurons and not in others, or might display distinct, cell-specific interactions with guidance receptors.

An alternative but not mutually exclusive explanation, consistent with previous results, is that Trio and TIAM-1 affect distinct aspects of Rac function in axon guidance. It is becoming apparent that the role of Rac GTPases in axon pathfinding is complex. Rac GTPases clearly regulate cytoskeletal events in the growth cone in response to guidance cues. For example, in *C. elegans*, Rac GTPases engage the actin-nucleating Arp2/3 complex and the actin-binding protein UNC-115/abLIM to promote filopodial extension [19], [24], [41]. It is also clear that Rac GTPases (e.g. MIG-2/RhoG) control the accumulation of guidance receptors (UNC-40/DCC) at the cell surface via trafficking [55], [62]. Thus, Rac GTPases might act both upstream and downstream of guidance receptors (Figure 9B). This might also explain in part why the axon guidance defects of *mig-2; ced-10* doubles are more severe than *tiam-1* alone. Rac GTPases might affect multiple aspects of axon guidance, whereas TIAM-1 might specifically regulate them in downstream cytoskeletal effects.

Trio might be a GEF that controls Rac activity in receptor trafficking upstream of UNC-40/DCC, possibly in a feedback mechanism (Figure 9B). In other words, UNC-6/Netrin activation of UNC-40/DCC might alter UNC-40/DCC trafficking to the cell surface, mediated by UNC-73/Trio and Rac. While the studies of Trio and Rac function in flies and vertebrates discussed above [56], [57] did not specifically address UNC-40/DCC trafficking, each can be explained by Trio and Rac regulating cell surface accumulation of Frazzled/DCC and the chimeric Frazzled-Robo receptor, similar to UNC-73/Trio and VAB-8L in *C. elegans* [55], [62]. In our studies, the myristoylated UNC-40 cytoplasmic domain is likely trafficked to the cell surface by a mechanism that is distinct from the endogenous UNC-40 molecule and might be independent of UNC-73/Trio and Rac-regulated cell surface accumulation, explaining why *unc-73* did not suppress MYR::UNC-40. Thus, suppression of MYR::UNC-40 might target those molecules that act downstream of UNC-40/DCC but not those involved in it's trafficking (Figure 9B). Our studies showing that *tiam-1* suppressed MYR::UNC-40 indicate that TIAM-1 might regulate Rac GTPases specifically downstream of UNC-40/DCC to mediate cytoskeletal responses.

### Rho GTPases and Rho GEFs in axon guidance

Many studies have shown that Rac and CDC-42 GTPases control lamellipodia and filopodia formation, yet it has been unclear how these molecules interact to regulate protrusion. Our results indicate that they act in a linear pathway, with CDC-42 controlling Rac activity mediated by TIAM-1. However, the *ced-10; mig-*2 double mutant is much more severe than *tiam-1*, so it is likely that another molecule regulates MIG-2 and CED-10 in parallel to TIAM-1 in axon pathfinding, possibly another GEF.

Rho-family GTPases control many cellular functions, and it has been assumed that upstream regulators confer specificity to Rho GTPase activity. Indeed, genomes encode many GTP exchange factors for Rho GTPases (e.g. the *C. elegans* genome encodes 19 DH GEF proteins). Our results provide experimental evidence for this idea, as we have shown that the TIAM-1 GEF but not the UNC-73/Trio GEF controls Rac function downstream of UNC-6/Netrin signaling in protrusion. UNC-73/Trio might act upstream of UNC-40/DCC and regulate its trafficking [55], [62] (Figure 9B). Future studies will be aimed at understanding how UNC-40/DCC links to CDC-42 to regulate TIAM-1, as well as understanding the roles of Rho GTPases and GEFs in repulsive axon guidance in response to UNC-6/Netrin.

## Methods

### *C. elegans* genetics and culture

*C. elegans* handling and culture were performed using standard techniques [63]. All experiments were performed at 20°C. The *tiam-1(tm1556)* allele was provided by the National Bioresource Project for the Experimental Animal “Nematode *C. elegans*” (S. Mitani), and the *tiam-1(ok772)* allele was provided to us by the *C. elegans* Gene Knockout Consortium via the *C. elegans* Genetic Center (CGC). Polymerase chain reaction (PCR) was used to verify the homozygosity of *tiam-1(ok772)* and *(tm1556)* in strains. The following mutations and genetic constructs were used: *LGI: unc-73(rh40), tiam-1(ok772) and (tm1556), lqIs37[osm-6::cdc-42(G12V)]; LGII: cdc-42(gk388), mIn1,vab-1(dx31), juIs76[unc-25::gfp]; LGIV: ced-10(n1993), zdIs4[mec-4::gfp], lqIs3[osm-6::gfp]; LGV: lqIs123[osm-6::myr::unc-73(DHPH1)]; LGX: mig-2(mu28), unc-6(ev400), slt-1(eh15), lqIs2[osm-6::gfp]; LG?: lqIs125[osm-6::myr::unc-73(DHPH2)], lqIs131[mec-7::myr::unc-40(cyto)::gfp], lqIs134[osm-6::myr::unc-73(DHPH1 S1216F)], lqIs165[osm-6::myr::tiam-1(DHPH)], lqIs216[osm-6::myr::tiam-1(DHPH T516F)]*. Extrachromosomal arrays: *lqEx504-506, osm-6::myr::unc-73(DHPH1); lqEx509-510, osm-6::myr::unc-73(DHPH)2; lqEx517-518, osm-6::myr::unc-73(DHPH1 S1216F); lqEx559-561, osm-6::myr::tiam-1(DHPH); lqEx571-572, lqEx584, osm-6::myr::tiam-1(DHPH T516F)*.

*C. elegans* transformation was performed with standard methods by microinjection of DNA into the hermaphroditic syncytial germline [64]. Transgenes were integrated into the genome using trimethylpsoralen and standard techniques. RNAi was performed using standard feeding RNAi protocols and clones from the Ahringer RNAi library that were confirmed by PCR [65].

All micrographs were obtained on a Leica DMRE microscope with a Qimaging Rolera MGi EMCCD camera or a Qimaging Retiga CCD camera. Openlab and IPlab software were used to obtain images.

### Scoring of PDE, VD/DD, and AVM axon defects

Fluorescence microscopy was used to score axon pathfinding defects of fourth larval stage (L4) or young adult hermaphrodite animals expressing a green fluorescent protein transgene for specific cells. To score and visualize PDE axons, the integrated transgenes *lqIs2 X* and *lqIs3 IV [osm-6 promoter::gfp]* were used [41], [66]. To score and visualize VDs and DDs axons, the integrated transgene *juIs76 II [unc-25 promoter::gfp]* was used [67]. To score and visualize AVM axons, the integrated transgene *zdIs4 IV [mec-4 promoter::gfp]* was used.

#### PDE axon pathfinding

The cell bodies of the PDE neurons (PDEL and PDER) are situated in the posterior lateral post-deirid ganglion. PDEs extend an axon ventrally to the ventral nerve cord, which then bifurcates and extends anteriorly and posteriorly in the ventral nerve cord. If the axon failed to reach the ventral nerve cord or wandered beyond a 45° angle from a straight line ventrally from the cell body, it was considered mutant.

#### VD/DD axon pathfinding

A VD/DD commissural axon that failed to reach the dorsal nerve cord or that wandered laterally before reaching the dorsal nerve cord was considered mutant. The percent of animals with pathfinding defects was noted, and the percentage of defective axons was noted.

#### AVM axon pathfinding

The AVM cell body is situated laterally on the right side, in the anterior half of the body of the animal. The axon extends ventrally towards the ventral nerve cord, which then extends anteriorly until the first pharyngeal bulb. Similarly to PDE axons, if the axon failed to reach the ventral nerve cord or wandered beyond a 45° angle from a straight line ventrally, it was considered mutant. p-value significance was determined using Fisher Exact Analysis.

#### Ectopic PDE and AVM protrusions

Ectopic lamellipodial and filopodial protrusions were identified as any thin ruffle-like or finger-like protrusion from the cell body, which is normally smooth and round, or axon of the neurons.

### Transgenes for expression in *C. elegans*

All *C. elegans* expression plasmids utilized the standard Fire expression vectors with the *unc-54* 3′ UTR (A. Fire, Stanford University). Primer sequences used to amplify *C. elegans* genomic and cDNA sequences to construct these plasmids are shown in Figure S9. For each transgene, at least three independent transgenic lines were generated, each with similar results. A *tiam-1* transcriptional promoter fusion plasmid was generated by amplifying by PCR the *tiam-1* upstream promoter region, a 4793-bp region upstream of T21E12.2 (the entire upstream region to the next predicted gene, T21E12.3) (Figure 2), and placing it upstream of the *cyan fluorescent protein* (*cfp*) coding region. For cell specific expression of full length TIAM-1, the open reading frame of the yk370h9 *tiam-1* cDNA was amplified by PCR and fused to mCherry at the 3′ end to create a C-terminally tagged TIAM-1::mCherry molecule (Figure 5). *tiam-1::mCherry* expression plasmids contained the *osm-6* promoter (to drive expression in the PDEs) [66] and the *unc-25* promoter (VD/DDs) [67] in separate constructs. The yk730h9 cDNA contained errors relative to the reference gene models that resulted in frame shifts. These were repaired in the yk730h9 cDNA by site-directed mutagenesis. A G residue after base 1039 relative to the start codon in Figure S2 was removed, and the A residue at position 2478 was added (see Figure S2).

A *gfp::mig-2* fusion plasmid was constructed by placing *gfp* in frame after the N-terminal myristoylation sequence of *mig-2* and before the remaining genomic coding region, allowing for GFP tagging of both myristoylated and prenylated forms of MIG-2 (Figure 5). *gfp::ced-10* was constructed by placing *gfp* at the N-terminus as previously described [48] (Figure 5). *gfp::mig-2* and *gfp::ced-10* expression was driven by the *osm-6* or *unc-25* promoter.

A plasmid containing an activated *cdc-42(G12V)* genomic coding region under the control of the *osm-6* promoter was constructed using site directed mutagenesis as described previously for *ced-10(G12V)* [41]. A *myr::unc-40(cyto)* plasmid under the control of the *mec-7* promoter was constructed as described in [9] (Figure 8A). The *myr::GEF(DHPH)* plasmids with the *osm-6* promoter were used as means of overactivating the GEFs as previously described [42] (Figure 4A). Three distinct DHPH GEF domains were fused in frame to an N-terminal myristoylation sequence as described in [44] and to a C-terminal GFP: the DHPH region of TIAM-1 (residues 488–856 relative to the A isoform); the Rac-specific UNC-73 DHPH domain (DHPH1) (residues 1193–1527 relative to the F55C7.7A isoform); and the Rho-specific UNC-73 DHPH domain (DHPH2) (residues 1795–2129). The coding regions of transgenes were sequenced after PCR to ensure that no mutations had been introduced.

### Cell culture, transfections, and co-localization assays in NIH3T3 fibroblasts

For expression in NIH 3T3 cells, a *cmv::mCherry::tiam-1* N-terminal fusion construct was generated by placing the yk37h9 *tiam-1* cDNA open reading frame, fused N-terminally to *mCherry*, behind the CMV promoter in the pTriEx-mCherry vector (K. Hahn, University of North Carolina). *cmv::gfp::ced-10* was constructed using the *ced-10* cDNA open reading frame [48] and the pEGFP-C1 vector (Clontech).

NIH 3T3 mouse fibroblasts were grown in DMEM (Cellgro) supplemented with 10% Cosmic Calf serum (HyClone) and 1% penicillin-streptomycin (P/S) and maintained in 5% CO_2_ at 37°C. Cells were plated onto coverslips the day before transfection at a density of 200,000 cells per well in a 6-well plate. For co-localization assays, pEGFP-C1 (vector only, “GFP”), pEGFP-C1 expressing CED-10 (“GFP::CED-10”), pTriEx-mCherry (vector only, “mCherry”) and pTriEx-mCherry expressing TIAM-1 (“mCherry::TIAM-1”) were transiently transfected into NIH3T3 cells using GeneExpresso Transfection reagent (Lab Supply Mall) according to the manufacturer's specifications. To visualize subcellular protein localizations at high magnification, the NIH 3T3 cells grown on coverslips and transiently transfected were fixed in 3.7% formaldehyde for 1 h at room temperature. Coverslips were then washed three times in 1× Phosphate Buffered Saline (PBS), one time in ddH_2_O, and then mounted onto a glass slide using ProLong Gold antifade (Invitrogen/Molecular Probes) mounting medium. The cells were then visualized using compound fluorescence microscopy (a Leica DMRE microscope with a Qimaging Rolera MGi EMCCD camera was used along with the IPLab and ImageJ software).

### Mant-GTP based exchange assay

We amplified the DHPH domains of TIAM-1 (residues 488–856 relative to isoform A), placed it into the pET-28b vector (Novagen), which has a T7 IPTG-inducible promoter and a C-terminal 6HIS-tag, and transformed this construct into BL21(DE3) Gold competent cells (Agilent Technologies). For the TIAM-1[DH(T516F)PH] construct, we used site directed mutagenesis to make a point mutation in the wild-type construct using the Quikchange kit (Stratagene). We purified TIAM-1[DHPH]::6HIS using Ni-column based chromatography (Qiagen). For the mant-GTP assay, we used the RhoGEF Exchange Assay Biochem Kit (Cytoskeleton, Inc) and followed instructions in the kit. We used 12.5 µM of TIAM-1[DHPH] in these experiments.

N- methylanthraniloyl-GTP (mant-GTP) is a fluorescently labeled nucleotide analog that can be incorporated by small GTPases. Using a Varian Cary Eclipse fluorescent spectrophotometer, we measured the amount of mant-GTP incorporated by the GTPase due to the spectroscopic difference between bound and free mant-GTP (when in the GTPase binding pocket, mant-GTP increases its fluorescence). The GTPase (human Rac1, Cdc-42 or RhoA) was incubated in buffer and mant-GTP for five minutes, after which time solvent (control) or the GEF (TIAM-1[DHPH]) was added. Experiments were recorded for one half hour.

## Supporting Information

Figure S1 A model of the *tiam-1* locus. (A) Black boxes represent exons with coding region open reading frame, white boxes represent non-coding exons, SL1 indicates that an SL1 trans-spliced leader sequence was present in the cDNA sequence, and AAAAA indicates the presence of a poly-A tail in the cDNA sequence. The regions removed by the *ok772* and *tm1556* deletions are indicated. *ok772* is an 838-bp deletion a coupled to an 18-bp insertion (CTGTAACTTAACTGTAAC), and *tm1556* is an 851-bp deletion. The yk730h9 cDNA spanned the predicted gene models C11D9.1 and T21E12.2, indicating that these regions are part of the same transcription unit and thus represent a single locus, the *tiam-1* locus. The dashed lines are an expansion of the 3′ end of the locus showing the structures of three cDNAs sequenced. yk730h9 is the longest cDNA and spans the entire locus. An arrow points to an intron that is spliced out specifically in yk730h9. An SL1 trans-spliced leader sequence is present at the 5′ end of yk730h9, indicating that it is a full-length cDNA. yk1684a10 is a shorter cDNA with splice variation. An in-frame stop codon is present in the 5′ region of the cDNA sequence, indicating that this cDNA contains the entire coding potential of this isoform. yk762f12 also encodes a shorter cDNA with splice variation and contains an SL1 trans-spliced leader, indicating that it contains the complete coding potential for this isoform. The sequences of these cDNAs have been deposited in Genbank. (B) The predicted molecules encoded by the three cDNAs. yk730h9 encodes isoform A, which contains a predicted EVH1-like domain and PDZ-like domain in addition to the DH and PH domains. yk762f12 encodes isoform B, which contains the PDZ-like domain and the DH/PH domains. yk1684a10 encodes isoform C which contains a truncated PDZ-like domain and the DH/PH domains. The regions removed by the deletions *ok772* and *tm1556* are indicated.
(TIF)

Figure S2 The nucleotide and amino acid sequence of *tiam-1 A*. Open reading frame of *tiam-1 A* encoded by a repaired version of the yk730h9 cDNA is shown, with the amino acid sequence below. The yk730h9 cDNA contained errors relative to the reference gene models that resulted in frame shifts. These were repaired in the yk730h9 cDNA by site-directed mutagenesis. An extra G residue after base 1039 relative to the start codon in Figure S2 was removed. This extra G after base 1039 is immediately after the exonic 5′ splice site of the intron that is spliced out in yk730h9 but not in the other two cDNAs yk762H12 and yk1684a10 (see Figure S1). Additionally, an A residue at position 2478 was added. This A is missing in yk730h9 but is present in the other two cDNAs yk762H12 and yk1684a10. The predicted EVH1-like domain, PDZ-like domain, and DH/PH domains are shaded grey. A potential N-terminal myristoylation sequence is underlined. The nucleotides removed by *ok772* and *tm1556* are shaded black. These do not represent the precise breakpoints of the deletions, as three of four breakpoints are within introns.
(PDF)

Figure S3 BLASTP and ClustalW analysis of the TIAM-1 molecule. (A) BLAST alignments showing of the predicted EVH1-like regions of *C. elegans* TIAM-1. (Ce) (this work), Still life/Tiam1 from *Ascaris suum* (As) (Genbank: ADY42014), and Still Life/Tiam1 isoform E from *Drosophila melanogaster* (Dm) (Genbank NP_001097519). While BLASTP and CCD did not recognize the EVH1 domain of *Ce* TIAM-1, they did recognize a conserved region in *As* TIAM-1 as an EVH1 domain. Identities are indicated. (B) An analysis of the putative PDZ-like domain as described for the EVH1 domain in (A). (C) ClustalW alignments of the predicted EVH1 and PDZ domains of the same molecules in (A and B). Conserved identical residues are indicated in red and by asterisks (*), and conserved similar residues are indicated by colons (:).
(PDF)

Figure S4 VD/DD and AVM axon defects in *tiam-1* mutants. In all micrographs, anterior is to the left and dorsal is up. (A) A fluorescent micrograph of an AVM neuron of a wild-type animal visualized with *mec-7::gfp*. (B) An AVM neuron from a *tiam-1(ok772)* mutant with an ectopic neurite. (C) A VD or DD motor neuron visualized with *unc-25::gfp* branched prematurely in a *tiam-1(tm1556)* mutant (arrow). The scale bars represent 2 µm.
(TIF)

Figure S5 Independent MYR::DHPH lines display ectopic protrusions. The percentages of ectopic protrusions from the AVM neurons of different extrachromosomal transgenic lines are shown. The transgenes express myristoylated forms of the DHPH domains from UNC-73 and TIAM-1 as described in Results. Above the graph are depictions of the predicted molecule expressed from each transgene. An “X” through the DH domain indicates the transgene harbors the point mutation that abolished Rac GEF activity *in vitro*, represented as “(DEAD)” on the X axis (see Figure 3). Error bars represent 2× standard error of the proportion, and 100 animals were scored for each strain. A box around the extrachromosomal array name indicates the extrachromosomal lines that were selected for integration and use in the experiments in Figure 4D. p values are relative to the wild type UNC-73[DHPH1] and TIAM-1[DHPH, respectively.
(TIF)

Figure S6 TIAM-1::mCherry localized to the periphery of neuronal cell bodies. In all micrographs, anterior is to the left and dorsal is up. (A) two PDE neurons with TIAM-1::mCherry expression driven by *osm-6::gfp* (arrows). TIAM-1::mCherry is excluded from the nucleus and localizes to the periphery of the cell body. (B) TIAM-1::mCherry accumulation at the periphery of VD/DD motor neuron cell bodies (arrows), driven by the *unc-25* promoter. The outlines of cell bodies were traced (dashed line) to indicate peripheral localization of TIAM-1::mCherry. The scale bar represents 2 µm for all micrographs.
(TIF)

Figure S7 GFP::CED-10 and TIAM-1::mCherry accumulate to specific sites in NIH-3T3 cells. GFP::CED-10 and TIAM-1 localize to discrete regions of the peripheral plasma membrane (arrowheads in (A) and (B). GFP::CED-10 induced lamellipodial ruffles but TIAM-1::mCherry did not. Neither GFP nor mCherry alone localized to discrete regions of the cell ((C) and (D)) as did the tagged CED-10 and TIAM-1 molecules. The scale bar in (A) represents 10 µm for all micrographs.
(TIF)

Figure S8 A *tiam-1* genomic fosmid clone rescued suppression of CDC-42(G12V). (B) A graph charting the percentage of PDE axons with ectopic lamellipodial and filopodial protrusions (X axis) in different genotypes (Y axis). *[cdc-42(G12V)]* represents animals harboring a transgene that expresses activated *cdc-42(G12V)* driven by the *osm-6* promoter. *[tiam-1(+)]* represents a transgene composed of the genomic fosmid clone WRM0633ch01 that harbors a wild-type copy of *tiam-1*. At least 100 PDE neurons were scored for each genotype, and p value significance was determined using Fisher's Exact analysis. Error bars represent 2× standard error of the proportion in both directions.
(TIF)

Figure S9 Primer sequences used to amplify *C. elegans* genomic or cDNA fragments to construct expression plasmids. The fragment of genomic DNA or cDNA amplified is underlined. (+) indicates the sense strand primer relative to the gene, and (−) indicates the anti-sense strand primer relative to the gene. Spacers and restriction enzyme sites added to the ends of the primers to aid in cloning are in italics.
(PDF)
