# Supplementary figures and images for: The Rac GTP Exchange Factor TIAM-1 Acts with CDC-42 and the Guidance Receptor UNC-40/DCC in Neuronal Protrusion and Axon Guidance
Source: PLoS Genet. 2012 Apr 26;8(4):e1002665. doi: 10.1371/journal.pgen.1002665 (PMC3343084; doi:10.1371/journal.pgen.1002665)

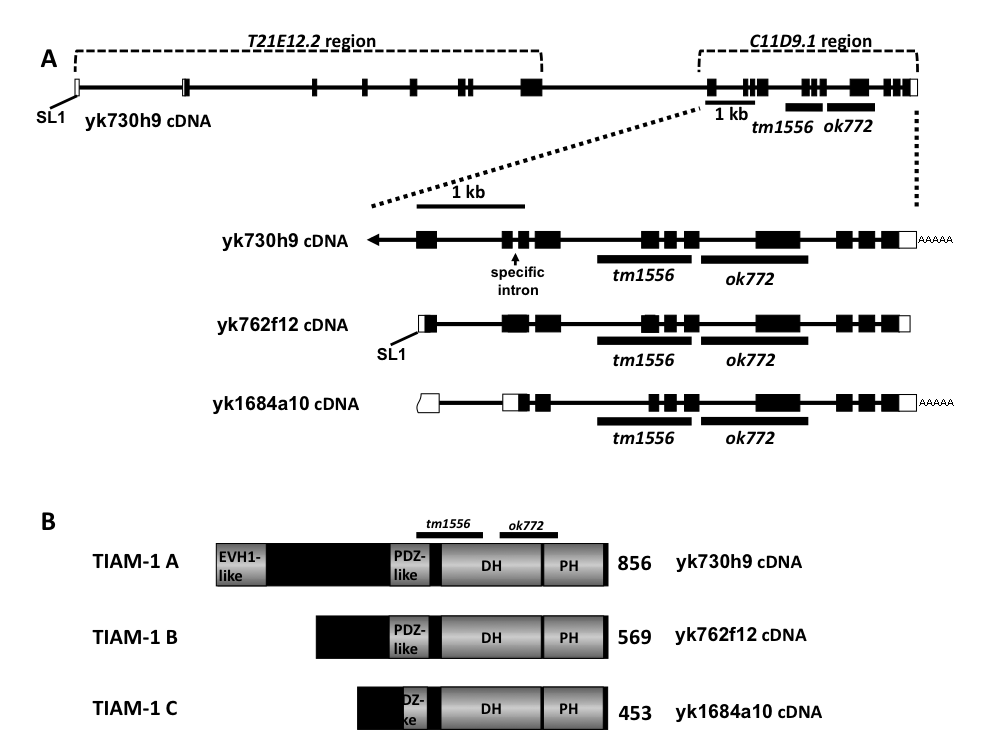

Supplement: Figure S1 — A model of the tiam-1 locus. (A) Black boxes represent exons with coding region open reading frame, white boxes represent non-coding exons, SL1 indicates that an SL1 trans-spliced leader sequence was present in the cDNA sequence, and AAAAA indicates the presence of a poly-A tail in the cDNA sequence. The regions removed by the ok772 and tm1556 deletions are indicated. ok772 is an 838-bp deletion a coupled to an 18-bp insertion (CTGTAACTTAACTGTAAC), and tm1556 is an 851-bp deletion. The yk730h9 cDNA spanned the predicted gene models C11D9.1 and T21E12.2, indicating that these regions are part of the same transcription unit and thus represent a single locus, the tiam-1 locus. The dashed lines are an expansion of the 3′ end of the locus showing the structures of three cDNAs sequenced. yk730h9 is the longest cDNA and spans the entire locus. An arrow points to an intron that is spliced out specifically in yk730h9. An SL1 trans-spliced leader sequence is present at the 5′ end of yk730h9, indicating that it is a full-length cDNA. yk1684a10 is a shorter cDNA with splice variation. An in-frame stop codon is present in the 5′ region of the cDNA sequence, indicating that this cDNA contains the entire coding potential of this isoform. yk762f12 also encodes a shorter cDNA with splice variation and contains an SL1 trans-spliced leader, indicating that it contains the complete coding potential for this isoform. The sequences of these cDNAs have been deposited in Genbank. (B) The predicted molecules encoded by the three cDNAs. yk730h9 encodes isoform A, which contains a predicted EVH1-like domain and PDZ-like domain in addition to the DH and PH domains. yk762f12 encodes isoform B, which contains the PDZ-like domain and the DH/PH domains. yk1684a10 encodes isoform C which contains a truncated PDZ-like domain and the DH/PH domains. The regions removed by the deletions ok772 and tm1556 are indicated. (TIF) [file pgen.1002665.s001.tif]

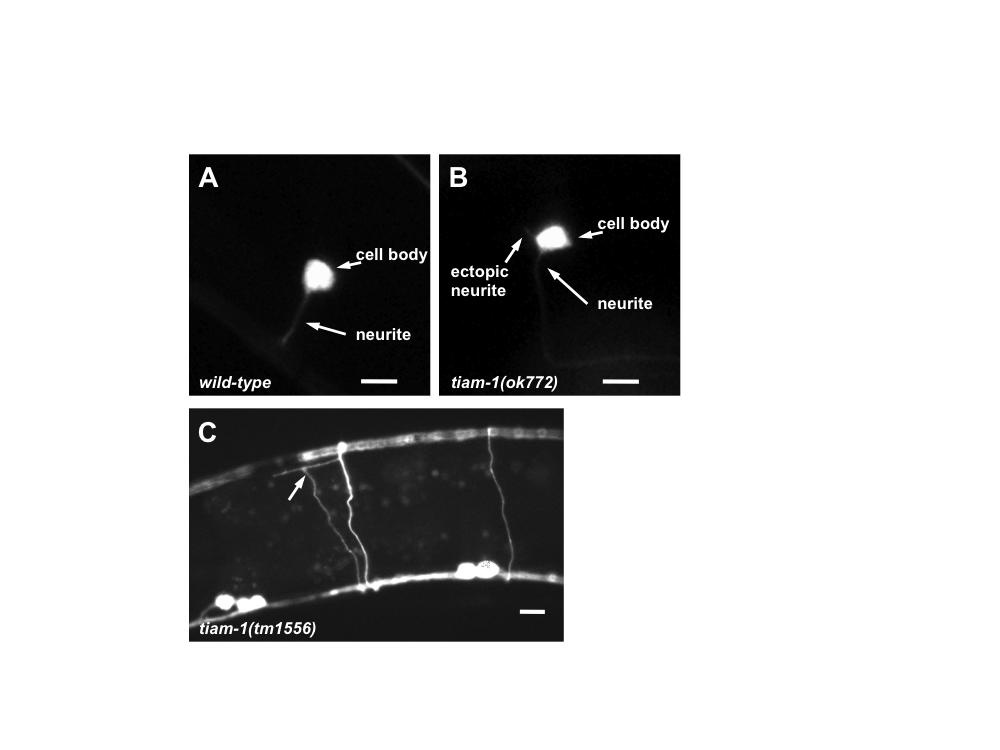

Supplement: Figure S4 — VD/DD and AVM axon defects in tiam-1 mutants. In all micrographs, anterior is to the left and dorsal is up. (A) A fluorescent micrograph of an AVM neuron of a wild-type animal visualized with mec-7::gfp. (B) An AVM neuron from a tiam-1(ok772) mutant with an ectopic neurite. (C) A VD or DD motor neuron visualized with unc-25::gfp branched prematurely in a tiam-1(tm1556) mutant (arrow). The scale bars represent 2 µm. (TIF) [file pgen.1002665.s004.tif]

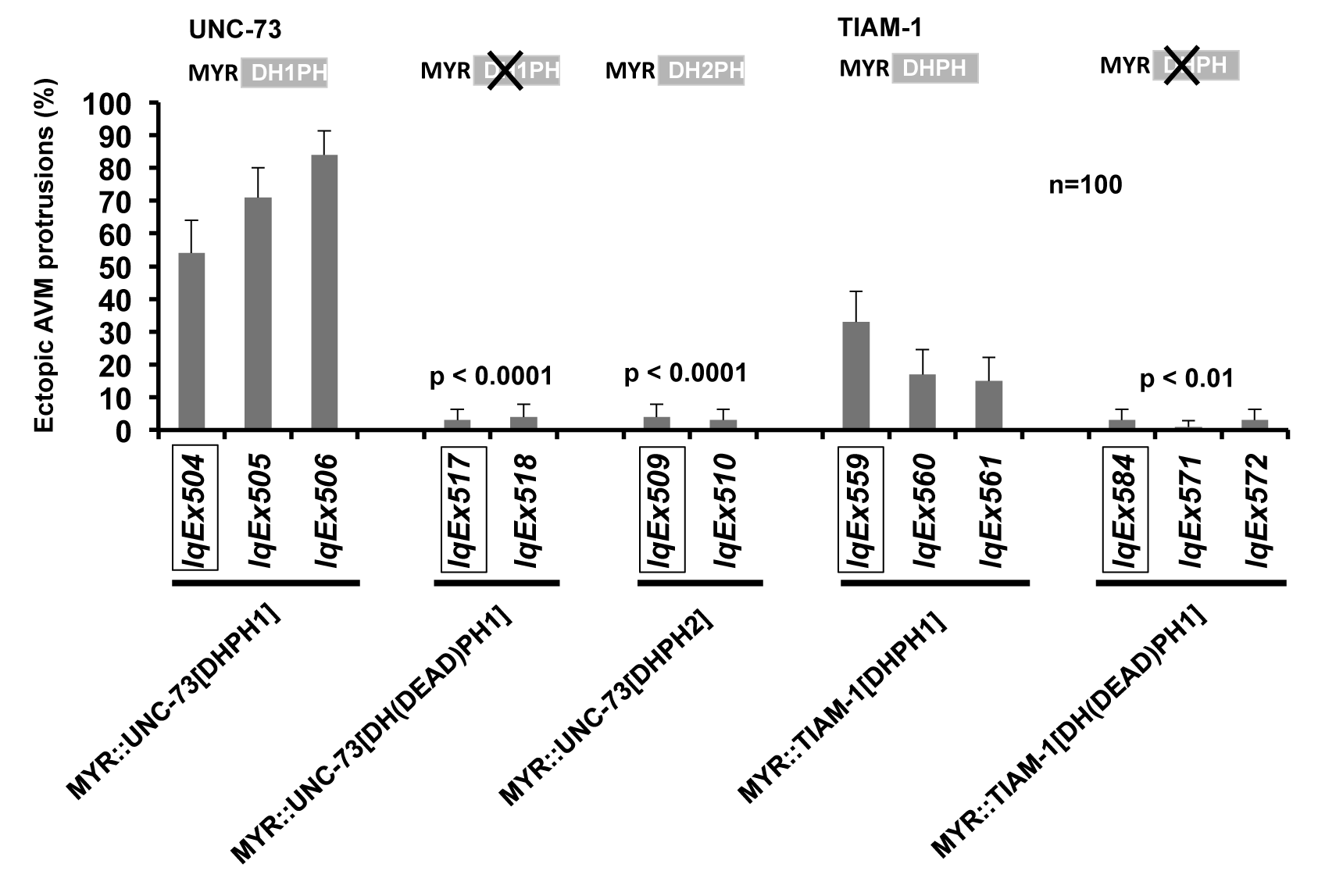

Supplement: Figure S5 — Independent MYR::DHPH lines display ectopic protrusions. The percentages of ectopic protrusions from the AVM neurons of different extrachromosomal transgenic lines are shown. The transgenes express myristoylated forms of the DHPH domains from UNC-73 and TIAM-1 as described in Results. Above the graph are depictions of the predicted molecule expressed from each transgene. An “X” through the DH domain indicates the transgene harbors the point mutation that abolished Rac GEF activity in vitro, represented as “(DEAD)” on the X axis (see Figure 3). Error bars represent 2× standard error of the proportion, and 100 animals were scored for each strain. A box around the extrachromosomal array name indicates the extrachromosomal lines that were selected for integration and use in the experiments in Figure 4D. p values are relative to the wild type UNC-73[DHPH1] and TIAM-1[DHPH, respectively. (TIF) [file pgen.1002665.s005.tif]

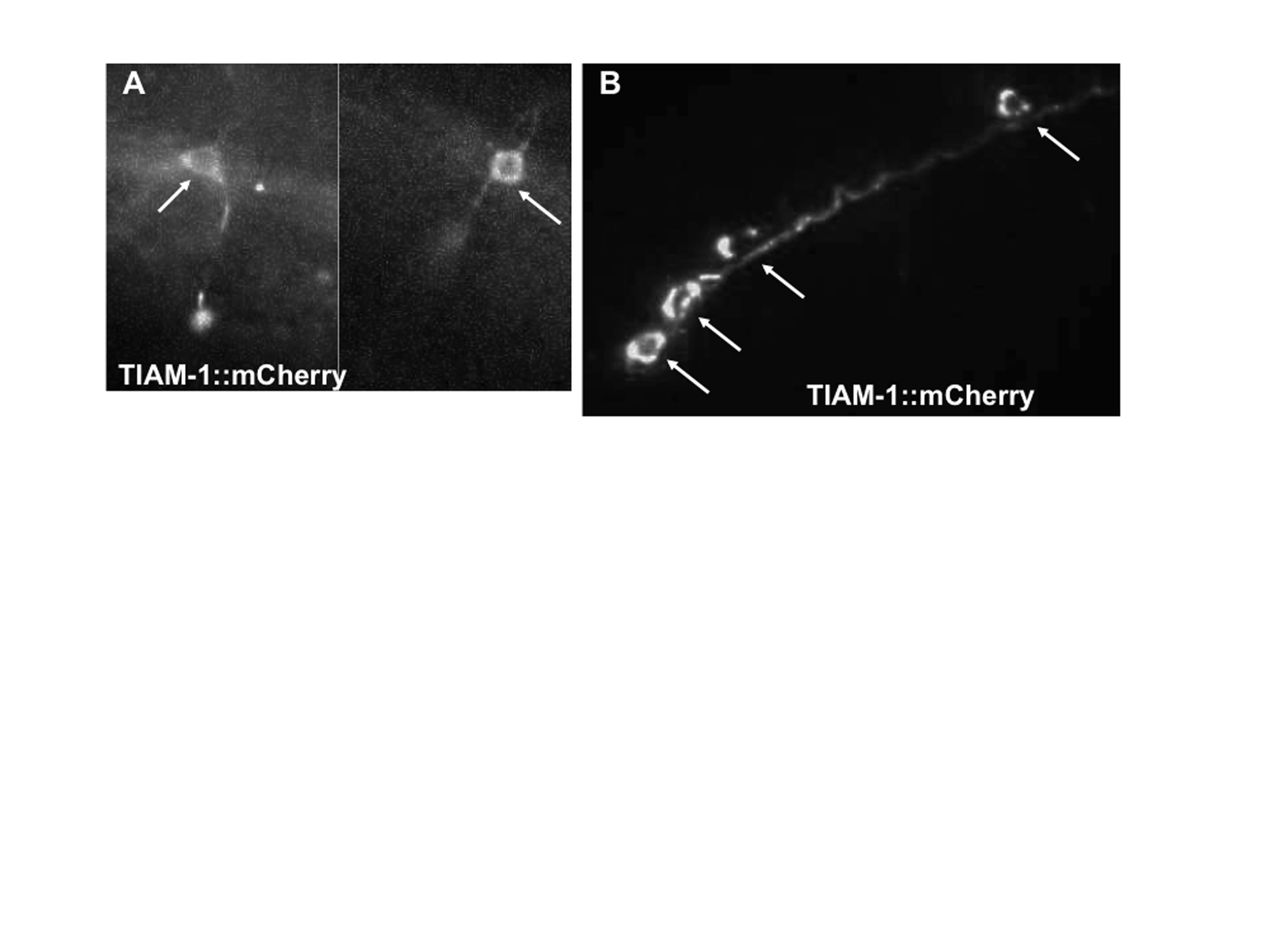

Supplement: Figure S6 — TIAM-1::mCherry localized to the periphery of neuronal cell bodies. In all micrographs, anterior is to the left and dorsal is up. (A) two PDE neurons with TIAM-1::mCherry expression driven by osm-6::gfp (arrows). TIAM-1::mCherry is excluded from the nucleus and localizes to the periphery of the cell body. (B) TIAM-1::mCherry accumulation at the periphery of VD/DD motor neuron cell bodies (arrows), driven by the unc-25 promoter. The outlines of cell bodies were traced (dashed line) to indicate peripheral localization of TIAM-1::mCherry. The scale bar represents 2 µm for all micrographs. (TIF) [file pgen.1002665.s006.tif]

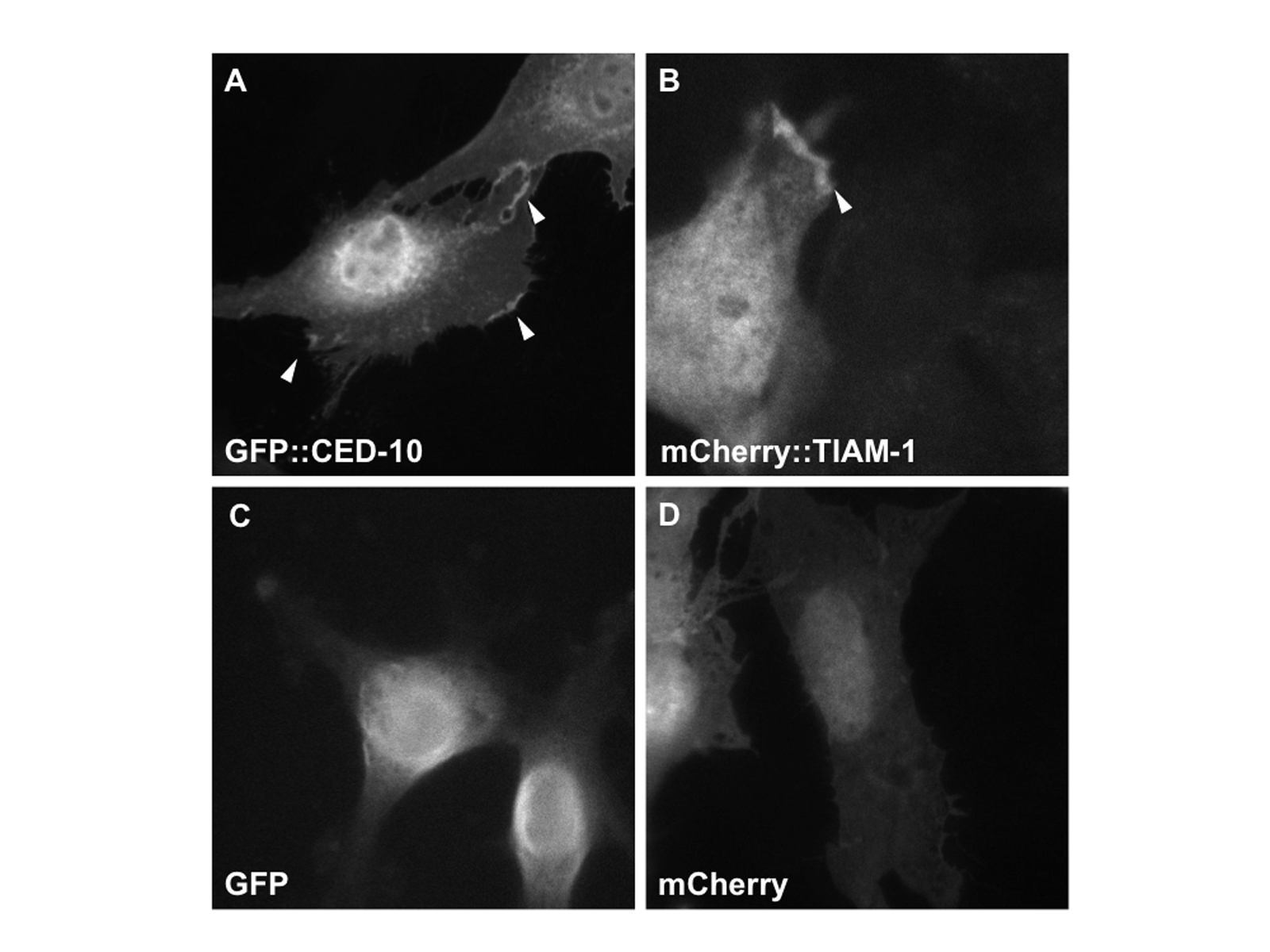

Supplement: Figure S7 — GFP::CED-10 and TIAM-1::mCherry accumulate to specific sites in NIH-3T3 cells. GFP::CED-10 and TIAM-1 localize to discrete regions of the peripheral plasma membrane (arrowheads in (A) and (B). GFP::CED-10 induced lamellipodial ruffles but TIAM-1::mCherry did not. Neither GFP nor mCherry alone localized to discrete regions of the cell ((C) and (D)) as did the tagged CED-10 and TIAM-1 molecules. The scale bar in (A) represents 10 µm for all micrographs. (TIF) [file pgen.1002665.s007.tif]

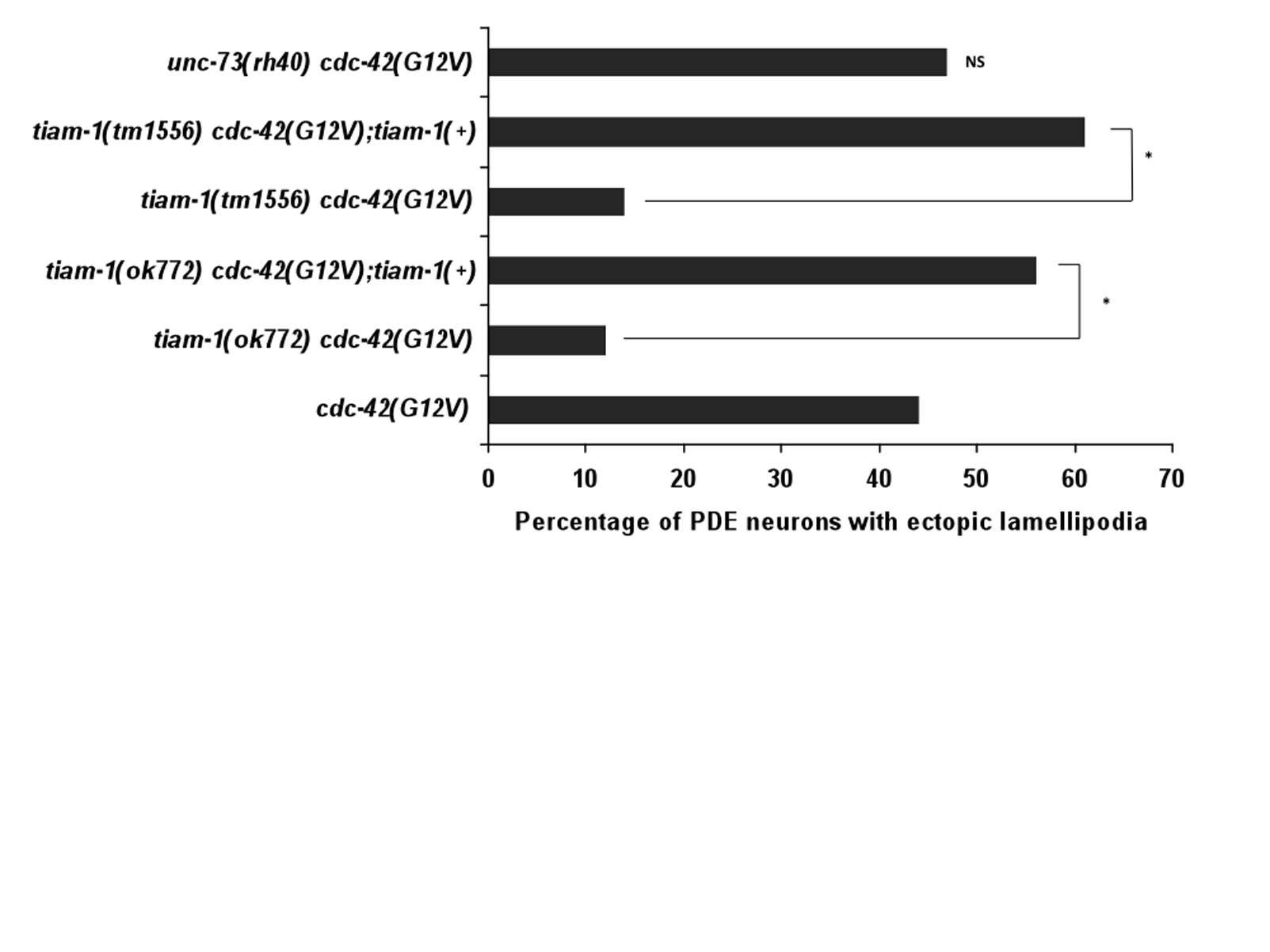

Supplement: Figure S8 — A tiam-1 genomic fosmid clone rescued suppression of CDC-42(G12V). (B) A graph charting the percentage of PDE axons with ectopic lamellipodial and filopodial protrusions (X axis) in different genotypes (Y axis). [cdc-42(G12V)] represents animals harboring a transgene that expresses activated cdc-42(G12V) driven by the osm-6 promoter. [tiam-1(+)] represents a transgene composed of the genomic fosmid clone WRM0633ch01 that harbors a wild-type copy of tiam-1. At least 100 PDE neurons were scored for each genotype, and p value significance was determined using Fisher's Exact analysis. Error bars represent 2× standard error of the proportion in both directions. (TIF) [file pgen.1002665.s008.tif]
