# Supplementary material for: The Rac GTP Exchange Factor TIAM-1 Acts with CDC-42 and the Guidance Receptor UNC-40/DCC in Neuronal Protrusion and Axon Guidance
Source: PLoS Genet. 2012 Apr 26;8(4):e1002665. doi: 10.1371/journal.pgen.1002665 (PMC3343084; doi:10.1371/journal.pgen.1002665)
Supplement: Figure S2 — The nucleotide and amino acid sequence of tiam-1 A. Open reading frame of tiam-1 A encoded by a repaired version of the yk730h9 cDNA is shown, with the amino acid sequence below. The yk730h9 cDNA contained errors relative to the reference gene models that resulted in frame shifts. These were repaired in the yk730h9 cDNA by site-directed mutagenesis. An extra G residue after base 1039 relative to the start codon in Figure S2 was removed. This extra G after base 1039 is immediately after the exonic 5′ splice site of the intron that is spliced out in yk730h9 but not in the other two cDNAs yk762H12 and yk1684a10 (see Figure S1). Additionally, an A residue at position 2478 was added. This A is missing in yk730h9 but is present in the other two cDNAs yk762H12 and yk1684a10. The predicted EVH1-like domain, PDZ-like domain, and DH/PH domains are shaded grey. A potential N-terminal myristoylation sequence is underlined. The nucleotides removed by ok772 and tm1556 are shaded black. These do not represent the precise breakpoints of the deletions, as three of four breakpoints are within introns. (PDF) [file pgen.1002665.s002.pdf]

```
   1 ATGGGCTCACGCCTCTCATGTTCTTGCTCACAAGCCGAATGCTGGACGAATGATGAATCT
   1  M  G  S  R  L  S  C  S  C  S  Q  A  E  C  W  T  N  D  E  S
            Myr?
  61 ACATTGAGAATTGACGCGGAGTTGTTTGAGTTATCACAGGACGGACAAAAGTGGGATAAG
  21  T  L  R  I  D  A  E  L  F  E  L  S  Q  D  G  Q  K  W  D  K

 121 CTTGACGGAAGGCTGGCCAACGTACGTGTATTCAACGCATTCGACGACTCTTCCCCACGT
  41  L  D  G  R  L  A  N  V  R  V  F  N  A  F  D  D  S  S  P  R

 181 CTTCTTGCCACATCATCATCCGGTCAAGTTCTTCTGGACACTCTAATTCCAATGGGAGAA
  61  L  L  A  T  S  S  S  G  Q  V  L  L  D  T  L  I  P  M  G  E    EVH1-like
                                                                    domain
 241 AAGGTTCATAAAGTCAGCGACTTTTTTGTGTATTTGAAGACGGAAGGAAGAACAATTGGA
  81  K  V  H  K  V  S  D  F  F  V  Y  L  K  T  E  G  R  T  I  G

 301 TTCAATACGTTAAGCTCGAGGGACACTTCCTTACTCATTTCTCAGGCGACAAATACAACT
 101  F  N  T  L  S  S  R  D  T  S  L  L  I  S  Q  A  T  N  T  T

 361 GCCTTCAATATGTTTCAACATTCATTCTCAGTTTCCGTATCCAGAATTGCTACATTTGAA
 121  A  F  N  M  F  Q  H  S  F  S  V  S  V  S  R  I  A  T  F  E

 421 GGTGATTCTATTCAACCTTGTGATGAGCAGCCAGAAACGTCAAATGTCTCACTAAATCCG
 141  G  D  S  I  Q  P  C  D  E  Q  P  E  T  S  N  V  S  L  N  P

 481 TTAAATATGGTTTTCAACTACAAAGGATCACCTGTTGAGATTGATTTGCTGGAATGTTTC
 161  L  N  M  V  F  N  Y  K  G  S  P  V  E  I  D  L  L  E  C  F

 541 GCGTGCTCATCAGAAGCTGAAAACTGTGTCGACATAGCACATGCCAACAACATATTTCGT
 181  A  C  S  S  E  A  E  N  C  V  D  I  A  H  A  N  N  I  F  R

 601 GTAATTGTGTCAGCACATGCTCATCTTTTTCTTATTCAAGCTCTGAATGTATCATCACTT
 201  V  I  V  S  A  H  A  H  L  F  L  I  Q  A  L  N  V  S  S  L

 661 CTTCTTGCTCGAACCACCTCGAATCTAGTTGCCATCAATTATTTGGCTCGGCAAAATGAT
 221  L  L  A  R  T  T  S  N  L  V  A  I  N  Y  L  A  R  Q  N  D

 721 AAAATTCAACAAAAATTGATTGCTCTGTTGGAAACATCAGAATCAAGTGAGAATGCGTCG
 241  K  I  Q  Q  K  L  I  A  L  L  E  T  S  E  S  S  E  N  A  S

 781 AAGGAAGATATTCGACAAATGCTTCTTCTGAAGAATGTAGTAGTGACACAGAGATTGAAC
 261  K  E  D  I  R  Q  M  L  L  L  K  N  V  V  V  T  Q  R  L  N

 841 TCACTTCAACAGAGAGCACTACCGGGAATTGAGCTTATCCAACACGATATCTCACCTCAA
 281  S  L  Q  Q  R  A  L  P  G  I  E  L  I  Q  H  D  I  S  P  Q

 901 ATTGATCAGAAGCTACTCGAAAAGTGTCGACTTCCATCACTGTTTTTATATCATTTTAAT
 301  I  D  Q  K  L  L  E  K  C  R  L  P  S  L  F  L  Y  H  F  N

 961 ATGAATTCGTTTCGAATATGGCGAAAACCAATGGAAACACGTGACATGAACACAATGGGA
 321  M  N  S  F  R  I  W  R  K  P  M  E  T  R  D  M  N  T  M  G

1021 TCATCAATGGCATCCACCAATCATGTGACAACTCATAATGGTACAACTACGTTTCATACT
 341  S  S  M  A  S  T  N  H  V  T  T  H  N  G  T  T  T  F  H  T

1081 ACGAATCCCACAAAACCAAGACTTTGTGAAGGATCGTTGGCTCCTGGATGCTCATTGGCC
 361  T  N  P  T  K  P  R  L  C  E  G  S  L  A  P  G  C  S  L  A

1141 GAGGCATCCGAACAGAGAAAACGAGAGATGGCGGAGCAGAAAAAAGAGTTGGCAGGCCCA
 381  E  A  S  E  Q  R  K  R  E  M  A  E  Q  K  K  E  L  A  G  P

1201 TCTTCCGCAGAGGATTACTTCGTCAGAAAGACAAATGGACGTCTTGGACTCACTATATAC
 401  S  S  A  E  D  Y  F  V  R  K  T  N  G  R  L  G  L  T  I  Y

1261 GCGCATAACGATGATGGGGTCATCAGGGCTGAGGTTCGTGGAGTGACGTCATTTGCTCCG
 421  A  H  N  D  D  G  V  I  R  A  E  V  R  G  V  T  S  F  A  P
```

```
1321 AGATGTGCTCAAGTTGGAGATTCGGTTGTAGCGGTAGACAGTGAACTCATATCATCAGTT
 441  R  C  A  Q  V  G  D  S  V  V  A  V  D  S  E  L  I  S  S  V

1381 AGAAACGCTTCGGATGTTGAGAAACTTTTGAGAATCGGAAAAGTAATACATTTACGAAGA
 461  R  N  A  S  D  V  E  K  L  L  R  I  G  K  V  I  H  L  R  R

1441 AAGACTCCTCTAACTCCAAGAGCACCGTCTCAACTCGACGCAACTAATGTCAATAAGAAA
 481  K  T  P  L  T  P  R  A  P  S  Q  L  D  A  T  N  V  N  K  K

1501 ACGGGCTGTGCAAAGTTGGCGATGGCTCTGCAAGAATTGTTGGTCACTGAGAAGAAATAT
 501  T  G  C  A  K  L  A  M  A  L  Q  E  L  L  V  T  E  K  K  Y

1561 GTCAGCGATCTTCGAGAGATGAATGAAGTTTTTCTGTGTATGAGACAAGTCCGAGATATT
 521  V  S  D  L  R  E  M  N  E  V  F  L  C  M  R  Q  V  R  D  I

1621 ATGAATGCAGCAATACGGTTGTATAAAATGCAAACATCGTTTGTGGACAGTATTGAGGAA
 541  M  N  A  A  I  R  L  Y  K  M  Q  T  S  F  V  D  S  I  E  E

1681 GCCATTGGAGACATGAGTAGACAGGATATTTCTGTTGCTCAGATTAGGGATTCTGTAATG
 561  A  I  G  D  M  S  R  Q  D  I  S  V  A  Q  I  R  D  S  V  M

1741 CGAGTGTGTGCAGTATTCATCAACAAATGTTCTGATTTCAAAATCTATGCAGAATATGCA
 581  R  V  C  A  V  F  I  N  K  C  S  D  F  K  I  Y  A  E  Y  A

1801 GCCGGTTACCATCTCCTCCAACATGAGATCAAAAACAAAAAAGAGCTTCTATCAAAATTG
 601  A  G  Y  H  L  L  Q  H  E  I  K  N  K  K  E  L  L  S  K  L

1861 GAAGCTGTAAACAGTACAAGAGAGCAACATTGTTCTTGGGAATCTCGGATGATTAAACCA
 621  E  A  V  N  S  T  R  E  Q  H  C  S  W  E  S  R  M  I  K  P

1921 GTCCAGAGAATTGTACAATATCCACTTTTACTGAAAAACATTGCCGATGCACTTCCGAAG
 641  V  Q  R  I  V  Q  Y  P  L  L  L  K  N  I  A  D  A  L  P  K

1981 GATGCACGAGAGAGGGTTCAAGTGGAGGCAGCATTGCAAAAAATGCAGACGTCTGCAGAA
 661  D  A  R  E  R  V  Q  V  E  A  A  L  Q  K  M  Q  T  S  A  E

2041 TATGTGAATGAAATGCAAAGGCTTCATGAAGATTATGCACAATATATGGAGACTGTTAGG
 681  Y  V  N  E  M  Q  R  L  H  E  D  Y  A  Q  Y  M  E  T  V  R

2101 AAAGCTAATGAGCCAATGTTGGCTGAAAAGAGTCTTCGCCTCGACACTCGAGAGCTTCTA
 701  K  A  N  E  P  M  L  A  E  K  S  L  R  L  D  T  R  E  L  L

2161 GTTTTTGCACACATAAAATGGAGAGATGCTCCAGCGGAGCACTACGTTGTGTTCGTGTTT
 721  V  F  A  H  I  K  W  R  D  A  P  A  E  H  Y  V  V  F  V  F

2221 CACTCTTTAATACTGCTCCTTCCATCGTATGCTAGGAAAGAGATTAAGATGAAATGGACT
 741  H  S  L  I  L  L  L  P  S  Y  A  R  K  E  I  K  M  K  W  T

2281 CGTGTCCTCCCAATAAATGAAGTTGATATCAACGAAATGCCAAATGATGCATTAAATCTA
 761  R  V  L  P  I  N  E  V  D  I  N  E  M  P  N  D  A  L  N  L

2341 AAACTTTATCATGCTGCTTTTGAAGGACCAAATGGACAATTAAGTCATTCCAATCCAAAT
 781  K  L  Y  H  A  A  F  E  G  P  N  G  Q  L  S  H  S  N  P  N

2401 ACAGTCTACAATATTGAATGTTGTCAATCTCAACTGAAACAGCATCTTATCAAAAATATC
 801  T  V  Y  N  I  E  C  C  Q  S  Q  L  K  Q  H  L  I  K  N  I

2461 AAAAAAGCTCGAACTTTATTCTCTCGAGAATCACATCGTCCATTAAGTGGATCTAGTCAA
 821  K  K  A  R  T  L  F  S  R  E  S  H  R  P  L  S  G  S  S  Q

2521 TCGGATGGTGGTTATGTGTCAGAAGTCTCTAAGGAACATCGAAATTCAAAATAG
 841  S  D  G  G  Y  V  S  E  V  S  K  E  H  R  N  S  K  *
```

PDZ-like domain

removed by *tm1556*

DH domain

removed by *ok772*

PH domain
