# Supplementary material for: The Rac GTP Exchange Factor TIAM-1 Acts with CDC-42 and the Guidance Receptor UNC-40/DCC in Neuronal Protrusion and Axon Guidance
Source: PLoS Genet. 2012 Apr 26;8(4):e1002665. doi: 10.1371/journal.pgen.1002665 (PMC3343084; doi:10.1371/journal.pgen.1002665)
Supplement: Figure S3 — BLASTP and ClustalW analysis of the TIAM-1 molecule. (A) BLAST alignments showing of the predicted EVH1-like regions of C. elegans TIAM-1. (Ce) (this work), Still life/Tiam1 from Ascaris suum (As) (Genbank: ADY42014), and Still Life/Tiam1 isoform E from Drosophila melanogaster (Dm) (Genbank NP_001097519). While BLASTP and CCD did not recognize the EVH1 domain of Ce TIAM-1, they did recognize a conserved region in As TIAM-1 as an EVH1 domain. Identities are indicated. (B) An analysis of the putative PDZ-like domain as described for the EVH1 domain in (A). (C) ClustalW alignments of the predicted EVH1 and PDZ domains of the same molecules in (A and B). Conserved identical residues are indicated in red and by asterisks (*), and conserved similar residues are indicated by colons (:). (PDF) [file pgen.1002665.s003.pdf]

A

EVH1 domain comparisons (BLAST)

```
Ce         21  TLRIDAELFELSQDGQKWDKEVHPHLDGRLANVRVFNAFDDSSPRLLATSSSGQVLLDTL  80
                 R  AELF LS D   W     P L      V V               S      D L
As          3   VVRFWAELFQLSADRSQW-AILYPSL----LSVTVSEISRKRHAVHVTATSASRIIVDQL  57

Ce         81  IPMGEKVHKVSDFFVYLKTEGRTIGFNTLSSRDTSLLISQATNTTAFNMF  125
                     V  VS  F Y      T G N  S  D          T  F  F
As         58  LDSSSQVTRVSSCFAYWRHQCQTYGVNFVSAEDCDHFCELSMETSRFIKF  107
28/109 = 26% identity


As          5  RFWAELFQLSADRS---QWAILYPSLLSVTVSEISRKRHAV-HVTA--TSASRIIVDQLL  58
               R WAE F  SA       W      L  V    I        H TA       I    L
Dm         39  RLWAEVFHVSASGAGTVKWQQVSEDLVPVNITCIQDSPECIFHITAYNSQVDKILDVRLV  98

As         59  -DSSSQVTRVSSCFAYWRHQC—QTYGVNFVSAEDCDHFCELSMETSRFIKFGTTS  111
                         S CF YW      T G NF S  D   F E       F      S
Dm         99   QPGTRIGQASECFVYWKDPMTNDTWGLNFTSPIDAKQFRECCSPSFKFSRKASSS  153


Ce         21  TLRIDAELFELSQDGQ---KWDKEVHPHLDGRLANVRVFNAFDDSSPRLLATSSSGQVLL  77
                 R  AE F  S  G    KW   V   L     N               A  S     L
Dm         39  --RLWAEVFHVSASGAGTVKW-QQVSEDLVP--VNITCIQDSPECIFHITAYNSQVDKIL   93

Ce         78  DT-LIPMGEKVHKVSDFFVYLKTE--GRTIGFNTLSSRDTSLLISQATNTTAFNM----F  125
               D  L   G      S  FVY K      T G N  S  D             F
Dm         94  DVRLVQPGTRIGQASECFVYWKDPMTNDTWGLNFTSPIDAKQFRECCSPSFKFSRKASSS  153
28/109 = 26% identity
```

B

<u>PDZ domain comparisons (BLAST)</u>

```
Ce           405  DYFVRKTNGRLGLTIYAHNDDGVIRAEVRGVTSFAPRCAQVGDSVVAVDSELISSVPDZR  469
                   Y   KT   LGLTI A   D VI AEVR V        QVGD VV VD   IS
As           410  EYRIEKTDRSLGLTICARQVDDVIKAEVRAVRD-GTTMVQVGDTVVSVDEHPISEL----  464

Ce           470  EMOVEDBYOKRNASDVEKLLRIGKVIHLRRK  481
                              A  VE LLR      LRR+
As           465  ----------QTADQVETLLRSATALVLRRR  485
36/90 = 40% identity


As           410  -EYRIEKTDRSLGLTICARQVDDVIKAE-----VRAVRDGTTMVQVG----DTVVSVDEH  459
                   E          G    A               V  V D       G    D
Dm          1185  VELQRTTLEQMWGFSVEAELIENAERQDELCCYVSRVEDKSVAMHNGIIKGDEIMVINGA  1244

As           460  PISELQTADQVETLLRSATALVLRRR  485
                    S L      E L
Dm          1245  IVSDLDMM-YLESVLQ---------E  1260


Ce           405  DYFVRKTNGRL-GLTIYAHNDDGVIRAEVRGVTSFAPRCAQVGDSVVAVDSELISSVPDZ  468
                      R T     G    A       R           R   V D  VA     I    D
Dm          1185  VELQRTTLEQMWGFSVEAELIENAERQDE--LCCYVSR---VEDKSVAMHNGIIKG--DE  1237

Ce           469  REMVEDBYKRNASDVEKLLRIGKVIHLRRK 481
                               SD        V
Dm          1238  IMVINGAI---VSDLD-MMYLESVLQ---E 1260
15/90 = 17% identity
```

C

EVH1 ClustalW

```
Ce  TLRIDAELFELSQDGQ---KWDKEVHPHLDGRLANVRVFNAFD-DSSPRLLATS—SSGQ
As  VVRFWAELFQLSADRS---QWAILYP-----SLLSVTVSEISR-KRHAVHVTAT—SASR
Dm  --RLWAEVFHVSASGAGTVKWQQVSE-----DLVPVNITCIQDSPECIFHITAYNSQVDK
      *: **:*.:* .     :*           *  * :            :::   . .:

Ce  VLLDTLIPMGEKVHKVSDFFVYLKTEGR--TIGFNTLSSRDTSLLISQATNTTAFNMF———
As  IIVDQLLDSSSQVTRVSSCFAYWRHQCQ--TYGVNFVSAEDCDHFCELSMETSRFIKF———
Dm  ILDVRLVQPGTRIGQASECFVYWKDPMTNDTWGLNFTSPIDAKQFRECCSPSFKFSRKASSS
    ::   *:  . :: :.*. *.* :      * *.*  *. * . : . .  :  *
```

PDZ ClustalW

```
Ce  -DYFVRKTNGRLGLTIYAHNDDGVIRAEVRGVTSFAPRCAQVGDSVVAVDSELISSVPDZ
As  -EYRIEKTDRSLGLTICARQVDDVIKAEVRAVR-DGTTMVQVGDTVVSVDEHPISELQT-
Dm  VELQRTTLEQMWGFSVEAELIENAERQDELCCYVSRVEDKSVAMHNGIIKGDEIMVING-
     :    . :   *::: *.  :.. : :            .*.     :. . *  :

Ce  REMVEDBYKRNASDVEKLLRIGKVIHLRRK
As  -----------ADQVETLLRSATALVLRRR
Dm  ------------AIVSDLDMMYLESVLQE-
                  *. *        *:.
```
