# Supplementary material for: The Rac GTP Exchange Factor TIAM-1 Acts with CDC-42 and the Guidance Receptor UNC-40/DCC in Neuronal Protrusion and Axon Guidance
Source: PLoS Genet. 2012 Apr 26;8(4):e1002665. doi: 10.1371/journal.pgen.1002665 (PMC3343084; doi:10.1371/journal.pgen.1002665)
Supplement: Figure S9 — Primer sequences used to amplify C. elegans genomic or cDNA fragments to construct expression plasmids. The fragment of genomic DNA or cDNA amplified is underlined. (+) indicates the sense strand primer relative to the gene, and (−) indicates the anti-sense strand primer relative to the gene. Spacers and restriction enzyme sites added to the ends of the primers to aid in cloning are in italics. (PDF) [file pgen.1002665.s009.pdf]

tiam-1 5' promoter region
(+) TIAM-1p-F
*CGCATGC*CAAGACTACTTTGGAAGTGTCC

(-) TIAM-1p-R
*CGCATGC*TCTGTAAAAAATATAAATTTTTAGTTGT

yk370h9 *tiam-1* cDNA ORF
(+) TIAM1-CHERRY F
*CCCTGCAG*GATGGGCTCACGCCTCTCATG

(-) TIAM1 PTN R BamHI
*CGGATCC*GGTTTTGAATTTCGATGTTCCTTAGA

gfp::mig-2
(+) mig-2 XmaImyrAgeI
*GCCCGGGATGTCTTCACCGTCGAGGACCGGT*CAGATCAAATGTGTAGTTGT
TGG

(-) mig-2 3'NheI
*GGCTAGC*TTACATAATATTGCAAGACTTCTTC

An *XmaI* fragment containing *gfp* coding region from vector pPD119.16 (A. Fire) was cloned into the *AgeI* site introduced by the mig-2 XmaImyrAgeI primer (underlined).

cdc-42 coding region
(+) cdc-42 5'
*GGCTAGC*GGCGGAATGCAGACGATCAA

(-) cdc-42 3'
*GGCTAGC*CTAGAGAATATTGCACTTCTTCT

cdc-42(G12V) site directed mutagenesis
(+) cdc-42 -G12V
AGTTTTACCGACAGCTACATCTCCAACGACGAC

(-) cdc-42 +G12V
GTCGTCGTTGGAGATGTAGCTGTCGGTAAAACT

<u>TIAM-1DHPH GEF</u>
(+) tiam-1 dh1
*GTCTAGA*GCACCGTCTCAACTCGACG

(-) tiam-1 dh2
*GTCTAGA*TTTTGAATTTCGATGTTCCTTAGA

<u>TIAM-1 DHPH T to F site directed mutagenesis</u>
(+) Tiam-1 DH/PH T-F 1
GCTCTGCAAGAATTGTTGGTCTTTGAGAAGAAATATGTCAGCGATCTTCGAG

(-) Tiam-1 DH/PH T-F 2
CTCGAAGATCGCTGACATATTTCTTCTCAAAGACCAACAATTCTTGCAGAGC

<u>UNC-73 DHPH GEF 1</u>
(+) unc-73DH1F
*GGCTAGC*GAGAAGAAGATCAATGAACGAC

(-) unc-73DH1R
*GGCTAGC*TCCAACTGAGTAAGCCTCCG

<u>UNC-73DHPH1 S to F site directed mutagenesis</u>
(+) unc-73DH1StoF+
CCAATGCGAGAGCTTATTCAATTCGAACGGGATTATATCAAAGAT

(-) unc-73DH1StoF-
ATCTTTGATATAATCCCGTTCGAATTGAATAAGCTCTCGCATTGG

<u>UNC-73 DHPH GEF 2</u>
(+) unc-73DH2F
*GGCTAGC*CCTGCAAAGGTGGAGAAGAC

(-) unc-73DH2R
*GGCTAGC*GTTGTATCGTCTCGGATCCAC

<u>TIAM-1 for mammalian cell culture</u>
(+) TIAM-1 CELL F1
*CAGATCT*CCATGTAGTAGTGACACAGAGATTG

(-) TIAM-1 CELL R1
*GGGATCC*TTATTTTGAATTTCGATGTTCCTTAGA

CED-10 for mammalian cell culture
(+) CED-10 CELL F1
*CGAATTC*ATGCAAGCGATCAAATGTGTCG

(-) [1]CED-10 CELL R1
*GGGATCC*TTAGAGCACCGTACACTTGCT
